# The freshwater snails (Gastropoda) of Iran, with descriptions of two new genera and eight new species

**DOI:** 10.3897/zookeys.219.3406

**Published:** 2012-09-03

**Authors:** Peter Glöer, Vladimir Pešić

**Affiliations:** 1 Biodiversity Research Laboratory, Schulstraße 3, D-25491 Hetlingen, Germany; 2 Department of Biology, Faculty of Sciences, University of Montenegro, Cetinjski put b.b., 81000 Podgorica, Montenegro

**Keywords:** Freshwater snails, checklist, new species, Iran

## Abstract

Using published records and original data from recent field work and revision of Iranian material of certain species deposited in the collections of the Natural History Museum Basel, the Zoological Museum Berlin, and Natural History Museum Vienna, a checklist of the freshwater gastropod fauna of Iran was compiled. This checklist contains 73 species from 34 genera and 14 families of freshwater snails; 27 of these species (37%) are endemic to Iran. Two new genera, *Kaskakia* and *Sarkhia*, and eight species, i.e., *Bithynia forcarti*, *Bithynia starmuehlneri*, *Bithynia mazandaranensis*, *Pseudamnicola georgievi*, *Kaskakia khorrasanensis*, *Sarkhia sarabensis*, *Valvata nowsharensis* and *Acroloxus pseudolacustris* are described as new to science; *Ecrobia grimmi* (Clessin & Dybowski, 1888), *Heleobia dalmatica* (Radoman, 1974) and *Hippeutis complanatus* (Linnaeus, 1758) are reported for the first time from Iran. Additional field work is highly desirable for a more appropriate evaluation of the extant freshwater snail biodiversity in Iran.

## Introduction

Considering the geographical position of Iran, a rich fauna of freshwater snails could be expected. A high level of endemism and a diverse mixture of Palaearctic and Paleotropical elements are characteristic of the Iranian freshwater fauna (Pešić and Saboori 2007).

Research of molluscs biodiversity in Iran has a relatively long tradition. In 1862, a group of Italian scientists undertook the first systematic expedition to Persia, which revealed a large number of molluscan samples. The results of this expedition have been published by Issel (1863). Two decades later, the mollusc fauna of the Caspian Sea was studied by Dybowski (1888). The first study on the molluscs diversity of inland water was done at the beginning of the XX^th^ Century by the Indian malacologists Annandale and his coauthors (Annandale and Prashad 1919, Annandale 1921, Annandale and Rao 1925) who studied the molluscan fauna of Seistan and Baluchistan Province. Biggs (1936, 1937, 1971) studied the malacofauna of the Central Plateau of Iran. In 1936 he noted: “Little has been written on the Mollusca of the Iranian Plateau. This was perhaps due to the inaccessibility of the interior in the past when the only method of travelling was by caravan”. Forcart (1935) studied molluscs from the Mazandaran Province. Starmühlner and Edlauer (1957) published the results of the Austrian Iran expedition of 1949/50 and 1956. Later on, Starmühlner (1961, 1965) studied molluscs from Northern and Eastern Iran collected by the Austrian A. Ruttner. More recently, Mansoorian (1986, 1994, 1998, 2000) published on the molluscan fauna of Iran.

However, our knowledge of freshwater snails of Iran remains scanty. Despite a growing number of data over the last years, resulting from the expeditions of the junior author in 2005, 2007, and 2011, literature records of freshwater snails in Iran have remained scattered and unreviewed, hampering ecological and biogeographical analysis. To what extent is the area of Iran unique and important for freshwater snail biodiversity? This paper attempts to answer such questions by compiling data on water molluscs and their current geographic distribution in Iran.

## Material and methods

The checklist of the freshwater snail fauna of Iran was compiled using published records and original data. The data from all publications were brought to the presently accepted state of taxonomy following Subba Rao (1989) (for Asian Fauna), Brown (1994) (for African Fauna) and Glöer (2002) (for the European Fauna), and papers published thereafter. Species referred to in postgraduate theses and scientific meetings are no formal publications and are consequently not considered herein.

During the field work, freshwater snails were collected by hand netting, sorted on the spot and preserved in 75 % alcohol. The data and locations of the sampling sites, where the junior author collected in 2005, 2007 and 2011 are listed in Appendix 1. In the section ‘New records’ collecting site abbreviations derive from the geographical database Pešić. The type material will be deposited in the Zoological Museum Hamburg (ZMH), Germany. Further, we had the opportunity to revise material of some Iranian freshwater snails deposited in the collections of the Natural History Museum Basel (NMB – Forcart’s collection), Zoological Museum Berlin (ZMB) and Natural History Museum Vienna (NHMW – Edlauer’s collection).

Not all species could be identified due to the sparsity of specimens and the non-characteristic shells, especially of small hydrobioid snails. Furthermore, the Caspian Sea fauna is not considered in the present paper. The order of families follows Bouchet and Rocroi (2005).

**Figure 1.**
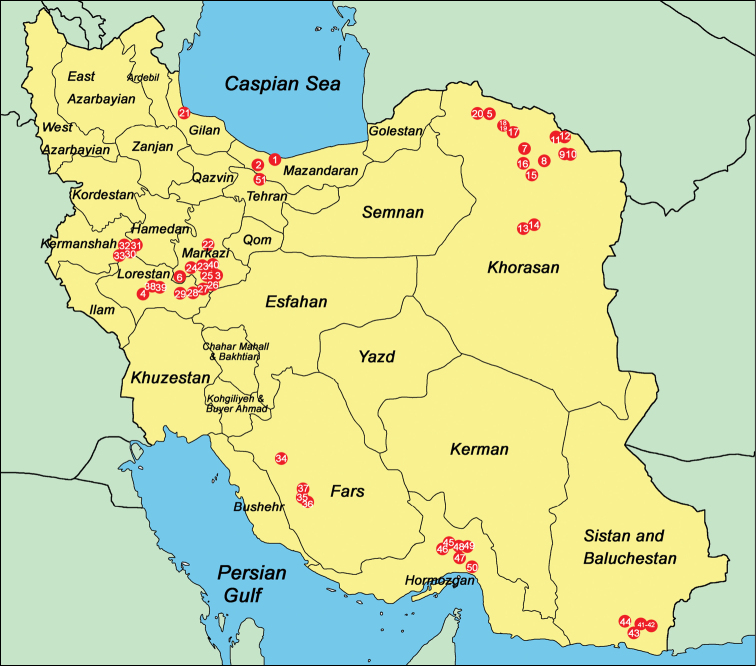
Map of Iran with dots showing the collection localities (corresponding to the sampling site numbers in Appendix). The total number of freshwater mollusc species collected from each province are as follows (in parentheses): Bushehr (1), Fars (15), Gilan (12), Hormozgan (13), Isfahan (10), Kerman (15), Hermanshah (4), Khorasan (5), Khuzestan (14), Lorestan (6), Markazi (5), Mazandaran (21), Qom (1), Seistan and Baluchestan (16), Semnan (1), Teheran (5), West Azarbayjan (1), Yazd (6), Zanjan (1).

## Results

### Systematics

#### Family Neritidae

Rafinesque, 1815

http://species-id.net/wiki/Neritidae

##### Remarks.

*Theodoxus* and *Neritina* are distinguished from each other by their ontogeny (Bandel 2001). While the *Theodoxus* species hatch from the spawn as miniature adult, *Nertina* species leave their spawn as planktotrophic larva that will float in the sea for a more or less extended period before its metamorphosis to a crawling young. However, at the adult stage the taxonomic separation of species of the genera *Theodoxus*and *Neritina*is not always easy. As most of the *Neritina* spp. are marine species and usually have a denticulate border of the columella and two apophysis of the operculum, most species of the genus *Theodoxus* are limnic and have a smooth border of the columella and one apophysis (the “rib”); some also have a small apophysis, the peg, on the operculum (Glöer 2002). Further, in *Neritina* the peg is thick and strong, while in *Theodoxus* it is, if exists at all, small and weak. A revision of this family, particularly its subdivion in clearly defined genera is needed.

### Genus *Neritina* Rafinesque, 1815

**Type species.** *Nerita pulligera* Linnaeus, 1758

#### Neritina mesopotamica

Martens, 1874

http://species-id.net/wiki/Neritina_mesopotamica

Figs 2a–c

##### Records from Iran.

Khuzestan Province (Mansoorian 2001).

##### Material examined.

Zoological Museum Berlin (ZMB), “*Neritina (Neritaea) anatolica* var. *mesopotamica*, Ras el Ain, Mesopot. Hausknecht”.

##### Remarks.

**Figure 2.**
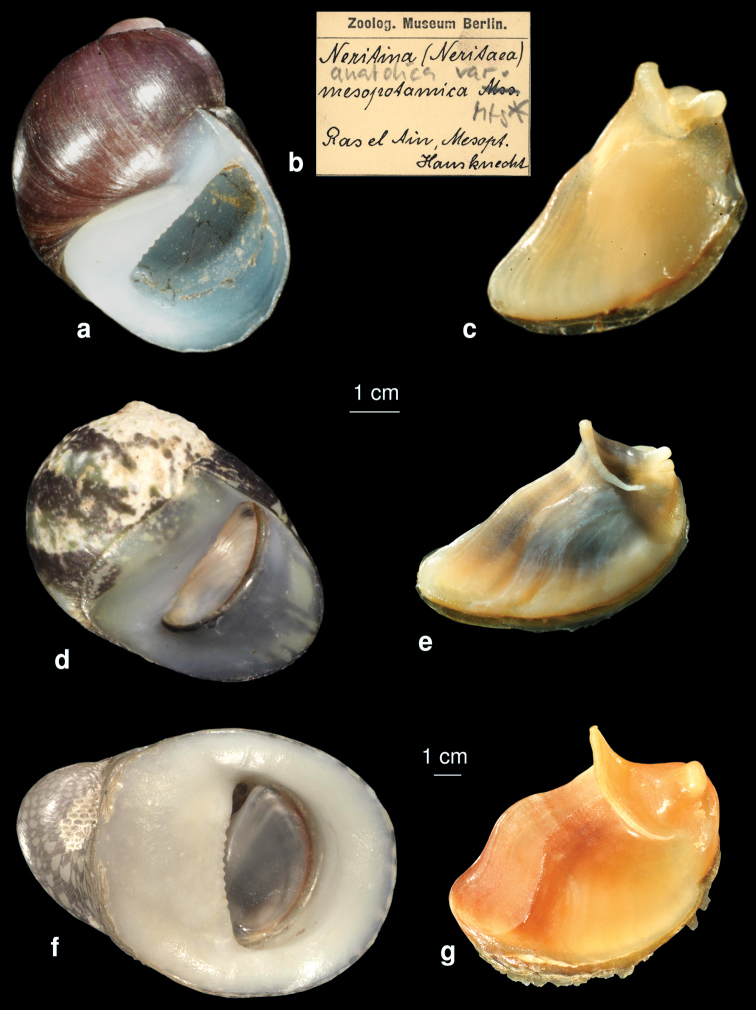
**a–c** *Neritina mesopotamica* **d–e** *Neritina euphratica* **f–g** *Neritina schlaeflii* **a** shell (syntype) **b** lable **c** operculum **d** shell (syntype, ZMZ 528916, Irak, Samava, photo: Eike Neubert) **e** operculum of *Neritina euphratica* from Euphrates **f** shell (syntype, ZMZ 529679, Persian Gulf, Island Ghaes, photo: Eike Neubert) **g** operculum of *Neritina schlaeflii* from Shatt Al-Arab-Fao region.

The height of the largest shell of the examined syntypes from Zoological Museum Berlin was 7 mm. Mansoorian (1994) in his identification key described shell of this species as being 14 mm high. Considering his photos (Mansoorian 1994), he probably confused it with *Neritina schlaeflii* Mousson, 1874 (Figs 2f–g).

##### Distribution.

Iraq, Iran (Khuzestan).

#### Neritina cinctellus

(Martens, 1874)

http://species-id.net/wiki/Neritina_cinctellus

Theodoxus cinctellus Martens, 1874. Syn.

##### Records from Iran.

Khuzestan Province (Chu et al. 1968, Massoud and Hedayeti-Far 1979).

**Remark.** According to the original description (Martens 1874) this species is characterized by the presence of denticulated border of the columella, and should be ascertained to the genus *Neritina*.

##### Distribution.

Iraq, Iran.

#### Neritina euphratica

Mousson, 1874

http://species-id.net/wiki/Neritina_euphratica

Figs 2d–e

##### Records from Iran.

Khuzestan Province (Massoud and Hedayeti-Far 1979, Mansoorian 2001).

##### Remark.

This speciesis characterized by a small shell with 6 mm in height and a small spire. The boder of the columella is straight and not denticulated. The operculum has a rib which is attenuated at its basis, the peg is thick and strong and split in two parts (fig. 2e).

##### Distribution.

Iraq, Iran.

### Genus *Theodoxus* Montfort, 1810

**Type species.** *Nerita fluviatilis* Linnaeus, 1758

#### Theodoxus fluviatilis

(Linnaeus, 1758)

http://species-id.net/wiki/Theodoxus_fluviatilis

Figs 3c 11a

Theodoxus doriae Issel, 1865 (synonymy)

##### Records from Iran.

(all mentioned as *Theodoxus doriae* Issel): Kerman (Issel 1863, Martens 1874, Biggs 1937); Gilan, Mazandaran and Lorestan Province (Mansoorian 2000).

##### New records.

Fars Province: IR13-07 [3 ex.]; IR14-07 [2 ex.]; Khorrasan Province: IR76-05 [1 ex]; IR 64-05 [1 ex.]; IR78a-05 [2 ex.]; IR79-05 [1 ex.]; Hormozgan Province: IR 17-11 [5 ex.]

##### Associated species.

*Melanopsis* sp.,* Radix* sp., *Planorbis intermixtus*, *Farsithyra farsensis*, *Physella acuta*.

##### Remarks.

Martens (1879) synonymised *Theodoxus doriae*, the species reported by Issel (1863) from S Iran, with *Theodoxus fluviatilis*. Later on, Mansoorian (2000) described the operculum of *Theodoxus doriae*, which has only a rib, no peg. However, the shell illustrated by Mansoorian (1994) agrees well with *Theodoxus fluviatilis*. Thus we follow Martens’ (1879) synonymisation of *Theodoxus doriae* with *Theodoxus fluviatilis*. Our samples revealed onlythe presence of *Theodoxus fluviatilis*.

##### Distribution.

W- to Central-Palaearctic. *Theodoxus fluviatilis* has been considered by many authors to be an exclusively European species (see e.g. Zhadin 1952, Glöer 2002). But Bourguignat (1864), Brown (1994) and Van Damme (1984) mentioned it from NW Africa (Morocco, Algeria). Records of this species in Turkey (Yıldırım 1994), and in Iran, confirm its wide distribution. However, it does not occur in Siberia (Vinarski, pers. comm.).

**Figure 3.**
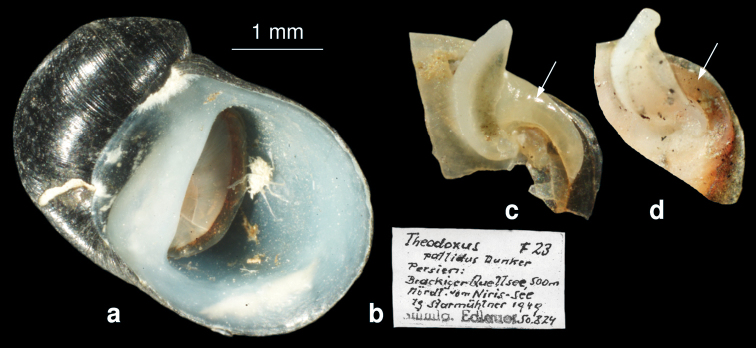
**a–c** *Theodoxus pallida* (from Edlauer’s collection, NHMW 75000/E/50824) **a** Shell with corroded apex **b** label of Edlauer’s collection **c** apophysis of *Theodoxus pallida* **d** apophysis of*Theodoxus fuiviatilis* (from IR79).

#### Theodoxus lituratus

Eichwald, 1838

http://species-id.net/wiki/Theodoxus_lituratus

##### Records from Iran.

Kerman Province (Biggs 1971); Mazandaran Province (Eichwald 1838, Eliazian et al. 1979).

##### Remarks.

This species has been described from the Caspian Sea. According to the original description (Eichwald 1838) this species is very distinct from the other *Theodoxus* spp. mentioned here.

##### Distribution.

Iran.

#### Theodoxus pallida

Dunker, 1861

http://species-id.net/wiki/Theodoxus_pallida

Figs 3a–b

##### Records from Iran.

Isfahan and Fars Province (Starmühlner and Edlauer 1957).

##### Material examined.

NHMW 75000/E/50824, “*Theodoxus pallidus* Dunker” Persien, Brackiger Quellsee, 500 m, nördl. vom Niris-see, leg. Starmühlner 1949.

##### Remarks.

Starmühlner and Edlauer (1957) provide a detailed description of the anatomy of this species but did not consider the operculum, the most important diagnostic feature. On the other hand, as figured in Starmühlner and Edlauer (1957), the receptaculum seminis and the bursa copulatrix differ in length (while being of equal length in *Theodoxus fluviatilis)*.

The re-examination of the specimens of *Theodoxus pallida* (Dunker, 1862) from Edlauer’s collection in NHMW clearly shows that this species is distinct from *Theodoxus fluviatilis* due to the shape of shell and the operculum (Fig. 3). As already mentioned by Dunker (1862) the spire in *Theodoxus pallida* is higher than in *Theodoxus fluviatilis*,and furthermore the apophysis of the operculum is broader and not attenuated at its basis (Fig. 3c). In addition the callus at border of the operculum in *Theodoxus pallida* is much stronger (Fig. 3c arrow).

##### Distribution.

Iran.

### Family Viviparidae J.E. Gray, 1847

Genus *Bellamya* Jousseaume, 1886

**Type species.** *Paludina bellamya* Jousseaume, 1886

#### Bellamya bengalensis

(Lamarck, 1822)

http://species-id.net/wiki/Bellamya_bengalensis

##### Records from Iran.

Khuzestan Province (Chu et al. 1968, Massoud and Hedayeti-Far 1979, Mansoorian 1994, 2001), Mazandaran Province (Mansoorian 2000).

##### Distribution.

According to Ramakrishna and Dey (2007) this species is widely distributed on the Indian subcontinent.

#### Bellamya hilmandensis

(Kobelt, 1909)

http://species-id.net/wiki/Bellamya_hilmandensis

##### Records from Iran.

Seistan and Baluchestan Province (Annandale et al. 1919).

##### Distribution.

Iran.

### Family Melanopsidae H. & A. Adams, 1854

#### Genus Melanopsis

Férussac, 1807

http://species-id.net/wiki/Melanopsis

##### Type species.

*Buccinum praemorsum* Linnaeus, 1758

##### Remark.

*Melanopsis praerosa* L. is a misspelling of *Melanopsis praemorsa* L.

#### Melanopsis costata

(Olivier, 1804)

http://species-id.net/wiki/Melanopsis_costata

Fig. 11e

##### Records from Iran.

Kerman Province(Martens 1874); Khuzestan Province (Prashad 1921, Chu et al. 1968, as *Melanopsis nodos*a: Massoud and Hedayeti-Far 1979, Mansoorian 2001).

##### New records.

Fars Province: IR13-07 [23 ad., 25 juv.].

##### Associated species.

*Farsithyra farsensis*.

##### Distribution.

Asia Minor, Syria, Palestine, Iraq, Iran.

#### Melanopsis doriae

Issel, 1865

http://species-id.net/wiki/Melanopsis_doriae

Fig. 4

##### Records from Iran.

Kerman Province (Issel 1863, Martens 1874, Biggs 1936, 1937, Starmühlner and Edlauer 1957, 1961, 1965); Fars Province (Starmühlner and Edlauer 1957); Yazd Province (Starmühlner and Edlauer 1957); Khuzestan Province (Mansoorian 1994, 2001); Mazandaran Province (Starmühlner and Edlauer 1957, Mansoorian 2000); Gilan Province (Starmühlner and Edlauer 1957); Bushehr Province (Starmühlner and Edlauer 1957).

##### New records.

Hormozgan Province: IR17-11 [2 ex.]; IR19-11 [1 ex.].

##### Material examined.

NHMW “*Melanopsis doriae* Issel” Persien, Kerman, aus teilweise eingestürztem Kanal, leg. Starmühlner 1949/50.

##### Associated species.

*Melanoides tuberculatus*, *Thiara scabra*, *Farsithyra farsensis*.

##### Remarks.

**Figure 4.**
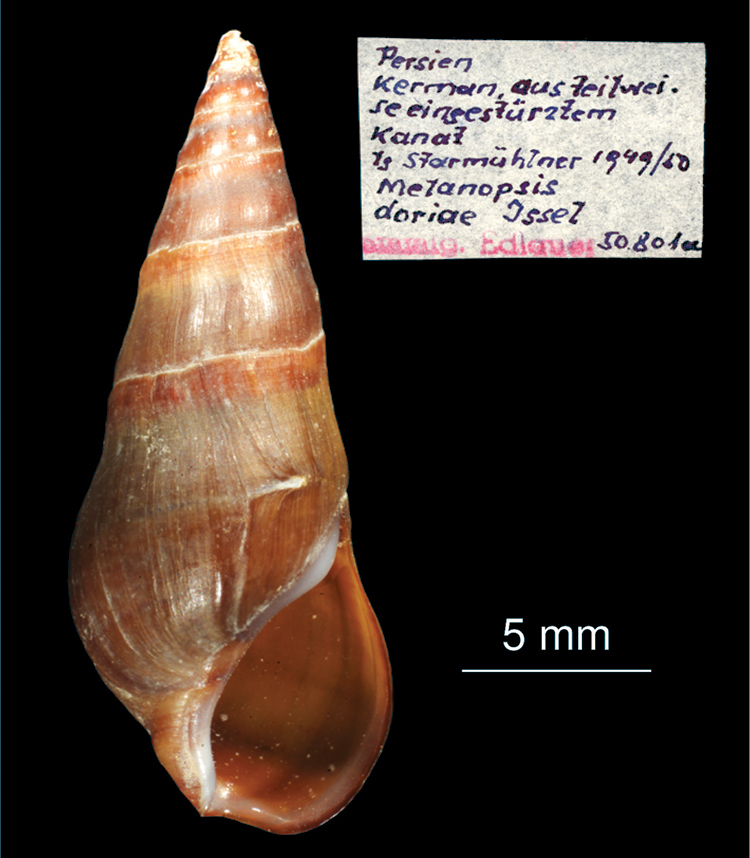
*Melanopsis doriae* (from Edlauer‘s collection, NHMW 750000/E/50801a): shell.

Starmühlner and Edlauer (1957) studied the anatomy of *Melanopsis doriae* and *Melanopsis kotschyi* showing differences in the nervous system. Furthermore they found differences in some features of the opercula between these species, and showed a strong morphological plasticity of the shells (see: Starmühlner and Edlauer 1957, plate 1: Figs g’, g’’, g’’’ and h’, h’’). Re-examintion of *Melanopsis doriae* from Edlauer’s collection in NHMW shows that the shell (Fig. 4) is slimmer than the shell of *Melanopsis* sp.

##### Distribution.

Iran.

#### Melanopsis kotschyi

Philippi, 1847

http://species-id.net/wiki/Melanopsis_kotschyi

##### Records from Iran.

Fars Province (Starmühlner and Edlauer 1957).

##### Remarks.

Seeremarks under previous species.

##### Distribution.

Iran.

#### Melanopsis sp.

Fig. 11d

##### Records from Iran.

Kerman Province (as *Melanopsis variabilis*:Martens 1874); Seistan and Baluchistan Province (as *Melanopsis deserticola*: Annandale and Prashad 1919); Isfahan and Yazd provinces (Biggs 1937); Fars province (as *Melanopsis buccinoidea variabilis*:Starmühlner and Edlauer 1957, as *Melanopsis praerosa*:Starmühlner 1961); Khuzestan Province (Chu et al. 1968, as *Melanopsis praerosa*:Massoud and Hedayeti-Far 1979, Manssorian 2001).

##### New records.

Mazandaran Province: IR02-05 [11 ad., 48 juv.]; Khorrasan Province: IR64-05 [12 ad., 39 juv.]; IR79-05 [3 ad., 4 juv.]; IR78a-05 [8 ad., 15 juv.]; IR78c-05 [2 ex.]; Fars Province: IR17-07 [2 ex]; Hormozgan Province: IR19-11 [21 ex.].

##### Associated species.

*Galba truncatula*, *Theodoxus fluviatilis*, *Planorbis intermixtus*, *Grossuana* sp., *Farsithyra farsensis*.

##### Remark.

The species of this genus have a high morphological plasticity and many species have been described. Glaubrecht (1993) tried to solve the complicated taxonomy by proposing to consider all circum-Mediterranean *Melanopsis* spp. as being part of one ‘superspecies’, *Melanopsis praemorsa*. However, we follow Neubert (1998) who believes that this approach does not solve the problem. In recent literature the ‘superspecies’ notion tends to be abandoned and the former species names are being reinstituted (see: Heller et al. 2005; Van Damme et al. 2010. This means that the smooth unsculptured species *Melanopsis praemorsa* sensu stricto (terra typica: Spain) is actually a western Mediterranean species and that unsculptured morphs from the Levant belong to other species, such as *Melanopsis buccinoidea*, *Melanopsis ammonis*, *Melanopsis dircaena*, *Melanopsis khabourensis* and *Melanopsis meiostoma* (Heller et al. 2005). Those from Mesopotamia have been described under *Melanopsis variabilis*, *Melanopsis deserticola*, *Melanopsis buccinoidea* and *Melanopsis praemorsa*. Further study is necessary to establish under which name or names the Iranian populations should be placed.

**Figure 5.**
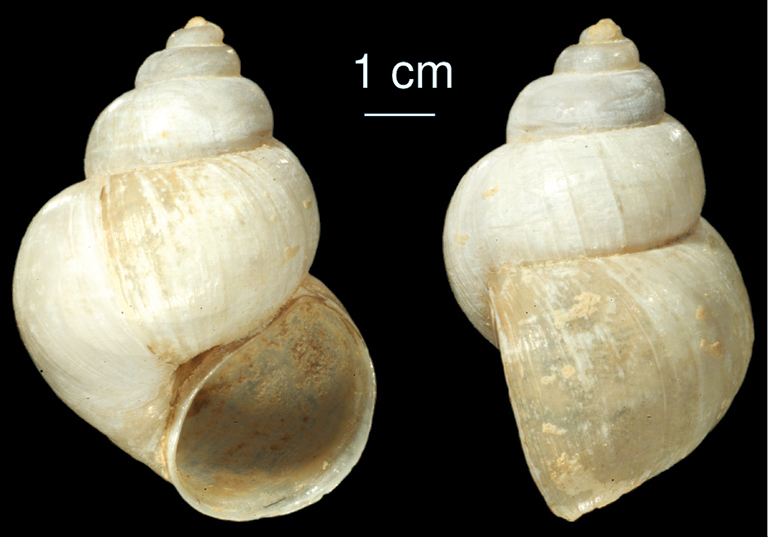
*Bithynia forcarti* sp. n. **a** shell,frontal view **b** shell,lateral view.

**Figure 6.**
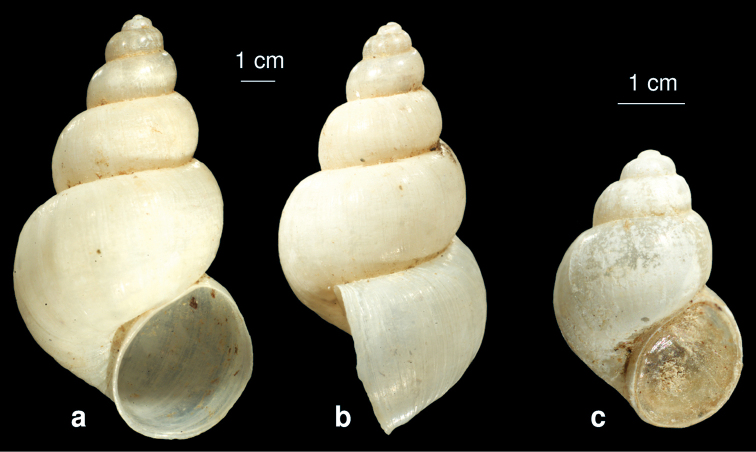
Shell of *Bithynia starmuehlneri* sp. n. **a** frontal view **b** lateral view **c** juvenile shell with operculum.

**Figure 7.**
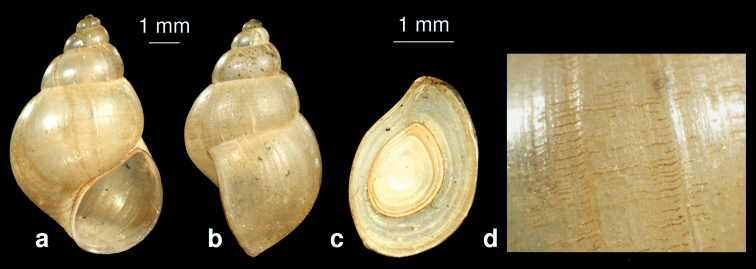
*Bithynia mazandaranensis*sp. n. **a, b** shell **c** operculum **d** detail of the shell surface.

### Family Potamididae H. & A. Adams, 1854

Genus *Cerithidea* Swainson, 1840

**Type species.** *Cerithium obtusum* Lamarck, 1822

#### Cerithidea cingulata

(Gmelin, 1790)

http://species-id.net/wiki/Cerithidea_cingulata

Fig. 8c

##### Records from Iran.

Hormozgan Province (Ghasemi et al. 2011).

##### New records.

Hormozgan Province:IR14-11 [21 ad., 6 juv.]; IR-20-11 [10 ex.].

##### Associated species.

*Ecrobia grimmi*, *Pseudamnicola* sp.

##### Distribution.

Indo-Pacific coast.

**Figure:**
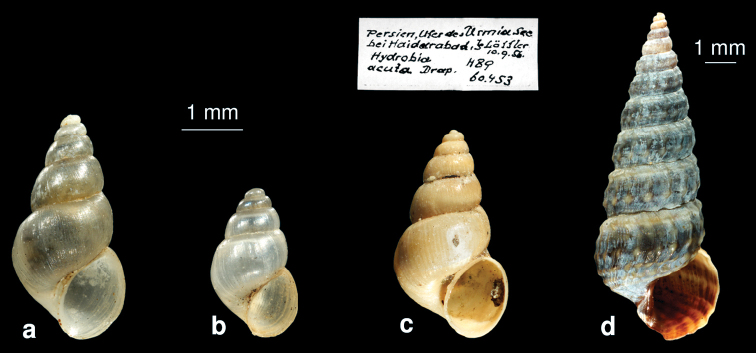
**Figure** 8**.** The molluscs of brackish waters. **a** *Ecrobia grimmi* **b** *Heleobia dalmatica* **c** *Ecrobia grimmi* from Edlauer‘s collection (NHMW, “*Hydrobia acuta*” 75000/E/60453) **d** *Cerithidea cingulata*.

**Figure 9.**
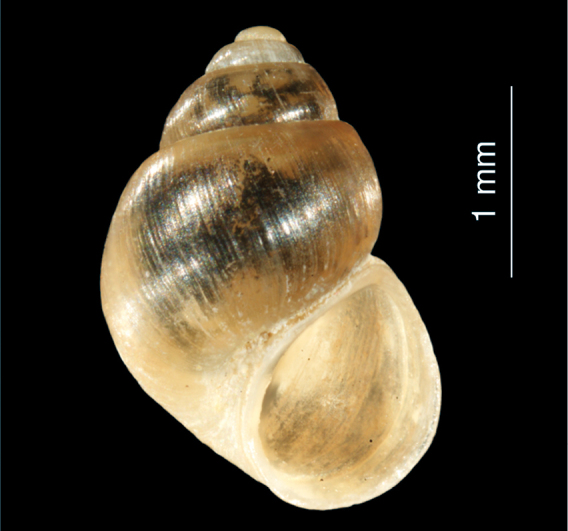
*Pseudamnicola georgievi* sp. n.: shell.

**Figure 10.**
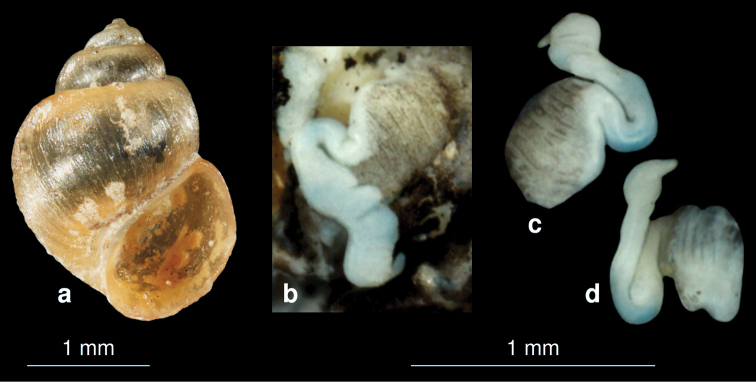
*Kaskakia khorrasanensis* sp. n. **a** shell **b** penis in situ **c–d** penis (c: dorsal view, d: ventral view).

### Family Thiaridae Gill, 1871

Genus *Thiara* Roeding, 1798

**Type species.** *Helix amarula*Linnaeus, 1758

#### Thiara scabra

(O.F. Müller, 1774)

http://species-id.net/wiki/Thiara_scabra

Fig. 12c

##### Records from Iran.

Seistan and Baluchestan Province (as *Melanoides scabra* var. *elegans*: Annandale and Prashad 1919); Isfahan Province (as *Melanoides scabra*: Biggs (1937); Hormozgan Province (Starmühlner and Edlauer 1957).

##### New records.

Hormozgan Province: IR08-11 [13 ex.]; IR17-11 [2 ex.].

##### Associated species.

*Farsithyra farsensis*, *Melanoides tuberculatus*, *Physella acuta*, *Melanopsis doriae*.

##### Distribution.

Indo-Pacific coasts.

**Figure 11.**
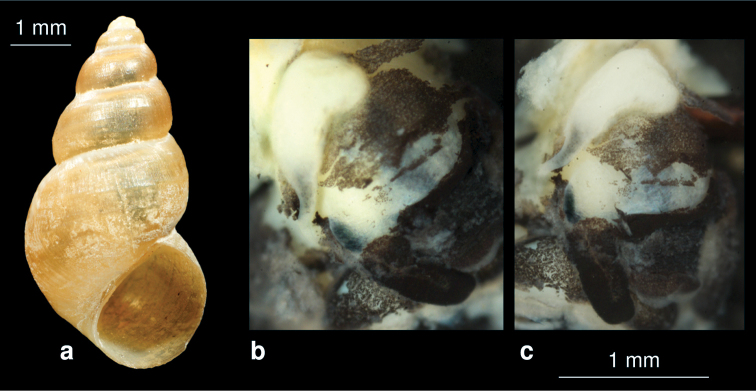
*Sarkhia sarabensis* nov. sp.**a** shell **b, c** penis in situ.

**Figure 12.**
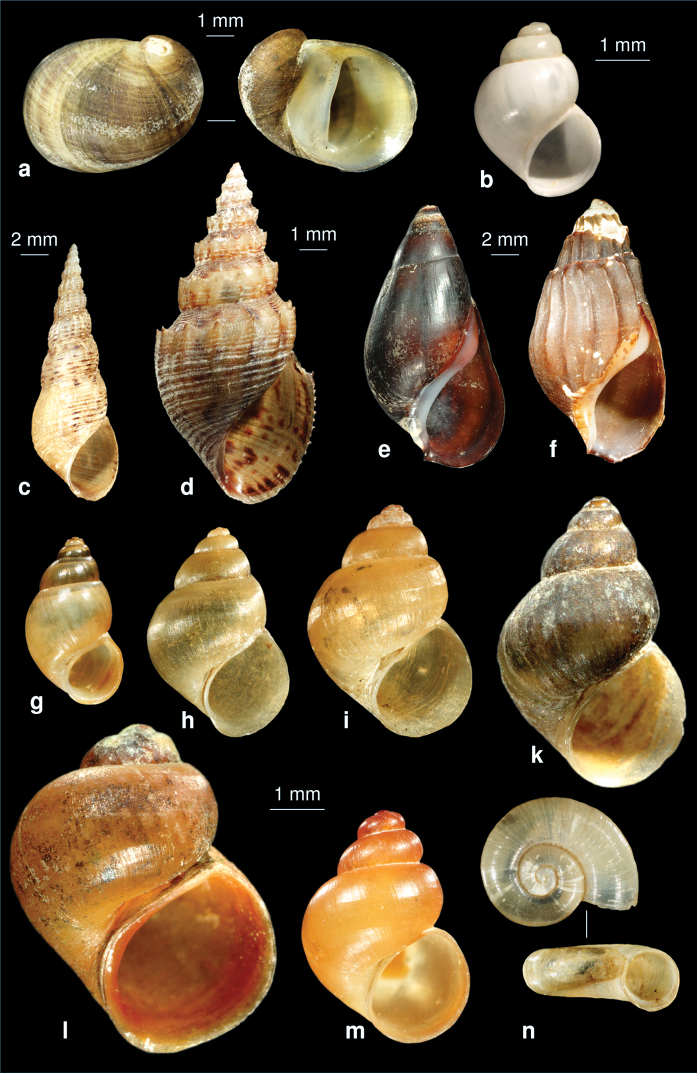
The prosobranch molluscs of Iran. **a** *Theodoxus fluviatilis* (operculum see Fig. 3d) **b** *Bithynia (Bithynia) ejecta* (syntype ZMZ 524006, Iraq, Samava, ex coll. Mousson, photo: E. Neubert) **c** *Melanoides tuberculatus* **d** *Thiara scabra* **e** *Melanopsis* sp. **f** *Melanopsis costata* **g** *Farsithyra farsensis* **h** *Sarkhia kermanshahensis*, **i:** *Pseudamnicola saboori* **k** *Pseudamnicola zagrosensis* **l** *Pseudobithynia irana* **m** *Pseudobithynia zagrosia* **n** *Valvata cristata*.

### Genus *Melanoides* Olivier, 1804

**Type species.** *Melanoides fasciolata* Olivier, 1804 = *Nerita tuberculata* O.F. Müller, 1774.

#### Melanoides tuberculatus

(O.F. Müller, 1774)

http://species-id.net/wiki/Melanoides_tuberculatus

Fig. 12b

##### New records.

Seistan and Baluchestan Province: IR8a-11 [5 juv.], IR8-11 [18 ex.]. Hormozgan Province: IR10-11 [3 ex.], IR17-11 [10 ad., 9 juv.], IR18-11 [1 ad., 8 juv.], IR19-11 [2 ex.].

##### Associated species.

*Melanopsis doriae*, *Thiara scabra*, *Farsithyra farsensis*.

##### Records from Iran.

Kerman Province (as *Melania tuberculata*: Issel 1863), Martens 1874, Biggs 1936, 1937, Starmühlner and Edlauer 1957); Seistan and Baluchestan Province (as *Melanoides pyramis*, *Melanoides tigrina*: Annandale and Prashad 1919, Biggs 1937); Hormozgan Province (Biggs 1937, Starmühlner and Edlauer 1957), (as *Melania tuberculata*: Starmühlner (1961); Isfahan Province (Biggs 1937); Yazd Province (Starmühlner and Edlauer 1957, as *Melania tuberculata*: Starmühlner 1965); Khuzestan Province (Chu et al. 1968, Mansoorian 2001); South Iran (Manssorian 1994); Fars Province (Starmühlner and Edlauer 1957): Mazandran Province (Starmühlner and Edlauer 1957, Mansoorian 2001).

##### Remarks.

The species *Melanoides pyramis* and *Melanoides tigrina*, which have been mentioned by Annandale and Prashad (1911) from Seistan and Baluchistan, have been listed by Westerlund (1886) as subspecies. However, due to the high morphological plasticity of *Melanoides tuberculatus* and in absence of any geographical seperation of these taxa, we list all *Melanoides* taxa under *Melanoides tuberculatus*.

##### Distribution.

S Asia, Arabia, Near East, Africa.

### Family Bithyniidae J.E. Gray, 1847

Genus *Bithynia* Leach, 1818

**Type species.** *Helix tentaculata* Linnaeus, 1758

#### Bithynia (Bithynia) tentaculata

(Linnaeus, 1758)

http://species-id.net/wiki/Bithynia_tentaculata

##### Records from Iran.

Mazandaran Province (Mansoorian 2000); Gilan and Lorestan Province (Mansoorian 2000).

##### Rejected records.

Mazandaran Province (Forcart 1935).

##### Remarks.

The Euro-Siberian species *Bithynia tentaculata* (Linnaeus 1758) has often been mentioned from Iran, Turkey and Greece. However, this species could not be found in Greece (Glöer et al. 2010) and probably does not occur in Turkey. The southern distribution border of this species lies possibly in N Bulgaria (Georgiev pers. comm.). An analysis of the specimens from NMB published by Forcart (1935) as *Bithynia tentaculata* shows that these specimens represent *Bithynia forcarti* sp. n. (see below). Thus, *Bithynia tentaculata* most probably does not occur in Iran and has been confused with *Bithynia forcarti* sp. n. or possibly with *Bithynia mazandaranensis* sp. n. (see below).

##### Distribution.

Euro-Siberian.

#### Bithynia (Bithynia) forcarti sp. n.

urn:lsid:zoobank.org:act:8A83711B-797D-4D86-99D5-72F217B14A89

http://species-id.net/wiki/Bithynia_forcarti

Figs 5a–b

##### Type locality.

Mazandaran Province, Tschalekuti.

##### Holotype

(NMB 11517a): shell height 7.5 mm, width 5.6 mm.

##### Paratypes.

Mazandaran Province, Tschalekuti (NMB 11517a, 26 ex.), Geniste d. Babul (NMB 11517b, 1 ex., NMB 11571c, 10 ex.)

##### Etymology.

Named after Lothar Forcart in appreciation on his studies of Iranian freshwater snails.

##### Description.

The whitish shell is conical with 5.5 whorls, which are convex with a deep suture and a small and acute apex. The convex whorls are flattened at the suture. The umbilicus is open. The aperture is ovate, angled at the top. The margin of the aperture is, from lateral view, slightly sinuated. The surface is smooth with fine growth lines. Shell height 5.5 – 7.5 mm, width 5.0 – 5.6 mm.

##### Differentiating features.

Due to theshape of the aperture (angled at the top), *Bithynia forcarti* sp. n. resembles *Bithynia mazandaranensis* sp. n. (see below). However, from the latter species it can be easily distinguished by the stepped whorls.

##### Remarks.

Formerly (Forcart 1935) this species has been confused with *Bithynia tentaculata*.

#### Bithynia (Bithynia) starmuehlneri sp. n.

urn:lsid:zoobank.org:act:5A63D216-B630-4808-8B2D-0F77E3EAE287

http://species-id.net/wiki/Bithynia_starmuehlneri

Figs 6a–c

Bulimus (Bithynia) leachi troschelii : Starmühlner and Edlauer 1957, non *troschelii* Paasch, 1842 (synonymy)

##### Type locality.

Border of Lake Urmia, W Azarbayian, 1949 leg. Starmühlner.

##### Holotype.

NHMW (50940): shell height 10.3 mm, width 5.6 mm.

##### Paratypes.

9 ex. from the type locality.

##### Etymology.

Named after Ferdinand Starmühlner, who collected this species in 1949.

##### Description.

The whitish shell is elongated conical with 6.5 whorls, which are convex with a deep suture and a small and acute apex. The umbilicus is open. The aperture is ovate. The margin of the aperture is, from lateral view, straight. The surface is smooth with fine growth lines. Shell height 8.2 – 10.3 mm, width 4.6 – 6.4 mm.

##### Differentiating features.

This slim species isthe largest *Bithynia* sp. known in Iran. It can be easily distinguished from the other *Bithynia* spp. by the larger dimensions of elongated shell with the stepped whorls and the not angled aperture.

##### Remarks.

This species has been misidentified by Starmühlner and Edlauer (1957) with *Bithynia troschelii*.

#### Bithynia (Bithynia) mazandaranensis sp. n.

urn:lsid:zoobank.org:act:22D0892E-8670-4131-9149-0F77C007BB94

http://species-id.net/wiki/Bithynia_mazandaranensis

Figs 7a–d

##### Type locality.

Mazandaran Province, Nowshahr city, pond near Caspian Sea, 51°31'E, 36°38'N, 18 June 2005.

##### Holotype

(ZMH 79369):Shell height 8.0 mm, width 5.0 mm.

##### Etymology.

Named after the region where the species was collected.

##### Description.

The horn-coloured shell is conical with 5.5 whorls, which are slightly convex with a clear suture and an acute apex. The umbilicus is closed. The aperture is ovate, angled at the top. The margin of the aperture is, from lateral view, sinuated. The surface bears a lattice structure. Shell height 8.0 mm, width 5.0 mm, aperture height 3.6 mm.

##### Differentiating features.

The new species resembles *Bithynia tentaculata* but differs from it by the following features: (i) the operculum is more angled (Fig. 7c), (ii) the whorls are more convex (Fig. 7a–b), and (iii) the surface has longitudinal and transverse striae (Fig. 7d).

##### Associated species.

*Planorbis carinatus*, *Anisus* sp., *Valvata cristata*, *Valvata nowshahrensis* sp. n., *Hippeutis complanatus*.

##### Remarks.

Probably this species formerly (e.g., Mansoorian 2000) was confused with *Bithynia tentaculata*. Because we had only an empty shell of this species, we do not know if it belongs to the genus *Bithynia* or *Pseudobithynia*, so our generic assignment is tentative. To address this question, anatomical studies of more specimens are necessary.

#### Bithynia (Bithynia) ejecta

Mousson, 1874

##### Records from Iran.

Isfahan Province – (as *Amnicola ejecta*: Biggs 1937).

##### Remarks.

Probably due to the small size of this species, Biggs (1937) assigned this species belongs to the genus *Amnicola*, although Mousson (1874) described it as a *Bythynia*, and pointed out that the operculum is characteristic for *Bythinia* and different from *Amnicola* (syn. to *Pseudamnicola*). Furthermore, Biggs (1937) found his species in the mountains, while the original description of *Bithynia ejecta* comes from the lowland, indicating the Biggs’s species is not conspecific with *Bithynia ejecta* and probably represents an undescribed species.

#### Bithynia (Bithynia) rubens

(Menke, 1830)

http://species-id.net/wiki/Bithynia_rubens

##### Records from Iran.

North Iran (Caspian Sea) – Eliazian et al. (1979).

##### Remarks.

This species could not be found in any of the neighbouring countries of Iran. Eliazian et al. (1979) don’t mention the source that led to their identification. The record and taxonomic status of this species is questionable and needs new confirmation.

#### Subgenus Gabbia

Tryon, 1865

Gabbia australis Tryon, 1865. Type species.

##### Remarks.

Some authors (e.g Subba Rao 1989, Nesemann et al. 2007) mention *Gabbia* as a genus. However, it seems not possible to distinguish the genera of the Bithyniidae by the shape of opercula (Mandahl-Barth 1968) and/or by shell forms, because these characters are found to be variable. On the other hand, the examined material of the family of Bithyniidae can be easily separated by the characteristics of penis morphology (having a penial appendix: *Bithynia* Leach 1818; or lacking a penial appendix: *Pseudobithynia* Glöer & Pešić 2006). In our study, we tentatively use the name *Gabbia* as a subgenus for small *Bithynia* species with a globular shell, originating from India.

#### Bithynia (Gabbia) sistanica

(Annandale & Prashad, 1919)

http://species-id.net/wiki/Bithynia_sistanica

##### Records from Iran.

Seistan and Baluchestan Province (as *Amnicola sistanica*: Annandale and Prashad 1919).

##### Remark.

Annadale and Prashad (1919) described this species as *Amnicola (Alocinma) sistanica* and depicted the penis morphology. Due to the presence of a penial appendix this species is ascertained to the genus *Bithynia*. The members of the genus *Pseudamnicola* (formerly *Amnicola*) have no penial appendix.

##### Distribution.

Iran; only known from N Seistan.

### Genus *Pseudobithynia* Glöer & Pešić, 2006

**Type species.** *Pseudobithynia irana* Glöer & Pešić, 2006

#### Pseudobithynia irana

Glöer & Pešić, 2006

http://species-id.net/wiki/Pseudobithynia_irana

Fig. 12k

##### Records from Iran.

Markazi and Lorestan Provinces (Glöer and Pešić 2006).

##### New records.

Lorestan Province:IR26-07 [10 ex.].

##### Associated species.

*Planorbis intermixtus*, *Radix* sp.

##### Distribution.

Iran; Markazi and Lorestan Provinces.

#### Pseudobithynia zagrosia

Glöer & Pešić, 2009

http://species-id.net/wiki/Pseudobithynia_zagrosia

Fig. 12l

##### Records from Iran.

Fars Province (Glöer and Pešić 2009).

##### Distribution.

Iran; known only from the locus typicus (Dasht Arzhan village, Shiraz to Kazerum road).

### Family Cochliopidae tryon, 1866

Genus *Heleobia* Stimpson, 1865

**Type species.** *Heleobia stagnorum* (Gmelin, 1791)

#### Heleobia dalmatica

(Radoman, 1974)

http://species-id.net/wiki/Heleobia_dalmatica

Fig. 8b

##### New records.

Hormozgan Province:IR14-11 [12 ad., 20 juv.].

##### Associated species.

*Cerithidea cingulata, *, *Ecrobia grimmi*, *Pseudamnicola sp*.

##### Remarks.

New for Iran.

##### Distribution.

Previously only known from the brackish part of rivers along the coast of Croatia (Radoman 1983).

### Family Hydrobiidae Stimpson, 1865

Genus *Hydrobia* Hartmann, 1821

**Type species.** *Cyclostoma acutum*Draparnaud, 1805

#### Hydrobia acuta

(Draparnaud, 1805)

http://species-id.net/wiki/Hydrobia_acuta

##### Records from Iran.

Isfahan Province (Biggs 1971).

##### Rejected records.

Fars Province (Starmühlner and Edlauer 1957).

##### Remark.

Probably this species has been confused with one of the following species (*Ecrobia grimmi*, *Heleobia dalmatica*), so all former records of this species in Iran are questionable. The record for this species is kept until the original material of Biggs could be studied.

### Genus *Ecrobia* Stimpson, 1865

**Type species.** *Turbo ventrosus* Montagu, 1803

#### Ecrobia grimmi

(Clessin & Dybowski, 1888)

http://species-id.net/wiki/Ecrobia_grimmi

Figs 8a, c

##### New records.

Hormozgan Province:IR14-11 [12 ad., 20 juv.].

##### Associated species.

*Cerithidea cingulata*, *Heleobia dalmatica*, *Pseudamnicola* sp.

##### Remarks.

On the base of molecular results, Haase et al. (2010) concluded that *Ecrobia grimmi* from the mixomesohaline Lake Sawa (Iraq) was possibly transported by migrating birds from the Caspian Sea. The identification of our material of *Ecrobia grimmi* as well of *Heleobia dalmatica* was confirmed by using molecular techniques (Martin Haase pers. communication). An analysis of the specimens from NHMW published by Starmühlner and Edlauer (1957) as *Hydrobia acuta* shows that these specimens probably belong to *Ecrobia grimmi* (see Fig. 8c).

##### Distribution.

Caspian Sea; Iraq, Iran.

### Genus *Pseudamnicola* Paulucci, 1878

**Type species.** *Bithynia lucensis* Issel, 1866

#### Pseudamnicola kotschyi

v. Frauenfeld, 1863

http://species-id.net/wiki/Pseudamnicola_kotschyi

##### Records from Iran.

Isfahan Province (Starmühlner 1961, 1965).

##### Distribution.

Iran: Isfahan Province; endemic.

#### Pseudamnicola saboori

Glöer & Pešić, 2009

http://species-id.net/wiki/Pseudamnicola_saboori

Fig. 12h

##### Records from Iran.

Khorasan and Markazi Provinces (Glöer and Pešić 2009).

##### Distribution.

Iran: Khorasan and Markazi Provinces.

#### Pseudamnicola zagrosensis

Glöer & Pešić, 2009

http://species-id.net/wiki/Pseudamnicola_zagrosensis

Fig. 12i

##### Records from Iran.

Kermanshah Province – Glöer and Pešić (2009).

##### Distribution.

Iran: Kermanshah Province.

#### Pseudamnicola raddei

Boettger, 1889

http://species-id.net/wiki/Pseudamnicola_raddei

##### Records from Iran.

Mazandaran Province – Forcart (1935).

##### Distribution.

Transcaspian region (Zhadin 1952).

##### Remarks.

In Russia it is listed as *Turkmenamnicola raddei* (Kantor et al. 2009).

#### Pseudamnicola georgiev sp. n.

urn:lsid:zoobank.org:act:D2E680D0-AAC4-45DF-954A-28D553EC957F

http://species-id.net/wiki/Pseudamnicola_georgievi

Fig. 9

##### Type locality.

Markazi Province, Ashtian to Arak road (ca. 5 km after Ashtian city, Ashtian county), 50°01'E, 34°34'N, ca. 1800 m asl., 21 June 2005.

##### Holotype

(ZMH 79370): Shell height 2.6 mm, width 1.9 mm.

##### Paratypes

(ZMH 79371): 6 ex. from type locality.

##### Etymology.

Named after Dr Dilian Georgiev in appreciation of his studies on Bulgarian hydrobiids.

##### Description.

The whitish shell is conical with 4.5 whorls, which are separated by a clear suture. The surface is glossy and finely striated. The apex is blunt, the umbilicus is closed, the aperture is ovate and pointed at the top. Shell height 2.4–2.6 mm, width 1.9 mm.

##### Differentiating features.

The conical shell with its pointed aperture (Fig. 9) clearly distinguished the new species from other Iranian members of the genus *Pseudamnicola*.

##### Remark.

We had only shells with dried tissue at our disposal. Since the penis morphology could not be examined, the assignment to the genus *Pseudamnicola* is provisional.

##### Distribution.

Iran; only known from the type locality.

#### Genus Kaskakia gen. n.

urn:lsid:zoobank.org:act:31BFCB62-BE86-43CE-A888-B0562CC2740E

http://species-id.net/wiki/Kaskakia

##### Diagnosis.

Shell conical. Penis broad at the basis, distal part with a bulbous and acute penis tip.

##### Type species.

*Kaskakia khorrasanensis* sp. n.

##### Etymology.

Named after the region where the species was collected.

##### Differential diagnosis.

The new genus appears to be close to *Pseudamnicola*, but can easily be distinguished by the unique morphology of the penis with bulbous and acute apex (vs. a broad elongated triangular penis in *Pseudamnicola*).

#### Kaskakia khorrasanensis sp. n.

urn:lsid:zoobank.org:act:8EDD45AD-46F2-4BC8-A7BE-73B44BBCDF6D

http://species-id.net/wiki/Kaskakia_khorrasanensis

Figs 10a–d

##### Type locality.

Khorrasan Province, Kaskak stream in Kaskak village, 59°10'E, 35°25'N, ca. 1800 m asl., 11 June 2005.

##### Holotype

(ZMH 79372): Shell height 2.5 mm, width 1.9 mm.

##### Paratypes

(ZMH 79373): 21 ex. from type locality.

##### Etymology.

Named for its occurrence in Khorrasan Province.

##### Description.

The yellowish shell is conical to globular with 5.5 whorls, which are slightly convex and separated by a clear suture (Fig. 10a). The whorls increase rapidly with a prominent body whorl. The surface is glossy and finely striated. The apex is acute, the aperture is ovate and angled at the top, the umbilicus is closed. Shell height 2.3–2.5 mm, width 1.8–1.9 mm.

##### Animal.

The mantle and head are black. The penis is broad at the basis and tapered at the distal end (Figs 10b–d).

##### Differentiating features.

As for the genus.

##### Distribution.

Iran: Khorrasan Province; known only from type locality.

#### Genus Sarkhia gen. n.

urn:lsid:zoobank.org:act:4AC287DC-4E88-4043-BA17-880E84883276

http://species-id.net/wiki/Sarkhia

##### Diagnosis.

Shell elongated conical. Penis simple, broad at the basis and tapered at the distal end, with a black pigmentation mark. The tentacles are cylindrical.

##### Type species.

*Sarkia sarabensis* sp. n.

##### Etymology.

Named after the region where the species was collected.

##### Differential diagnosis.

The genus seems to be closely related to *Pseudamnicola* (in the following, in parentheses), but theunique morphology of the penis, broad at the basis and tapered at the distal end (Figs 10b–c), with a black pigmentation mark (vs. broad and elongated triangular penis), and the presence of broad cylindrical tentacles (slim cylindrical tentacles) will separate the new genus from *Pseudamnicola*.

#### Sarkhia sarabensis sp. n.

urn:lsid:zoobank.org:act:F7FBD536-0970-4B9B-A0AC-EAF9E7C91C72

http://species-id.net/wiki/Sarkhia_sarabensis

Fig. 11a–c

##### Type locality.

Kermanshah Province, Sarabe–Sahne (= Sarabe – bede – Sarkh) city, stream, 27 June 2005.

##### Holotype

(ZMH 79374): Shell height 5.9 mm, width 2.3 mm.

##### Paratypes

(ZMH 79375): 1 specimen dissected.

##### Etymology.

Named after the region where the species was collected.

##### Description.

The yellowish shell is elongated conical with 6.5 whorls, which are slightly convex and separated by a deep suture. The aperture is oval with a sharp periostome, the umbilicus is closed. The surface is dull. Shell height 5.9 mm, width 2.3 mm.

##### Differentiating features.

The slim elongated conical shell with more than 5 whorls (Fig. 11a) is characteristic and separates this species from *Sarkhia kermanshahensis* (see below).

##### Distribution.

Iran, Kermanshah Province; only known from type locality.

#### Sarkhia kermanshahensis

(Glöer & Pešić, 2009) comb. n.

http://species-id.net/wiki/Sarkhia_kermanshahensis

Fig. 12g

Pseudamnicola kermanshahensis Glöer & Pešić, 2009 (synonymy)

##### New records.

Markazi Province: IR51 [2 ex.].

##### Records from Iran.

Kermanshah Province (as *Pseudamnicola kermanshahensis* Glöer and Pešić 2009).

##### Remarks.

This species has originally been placed in the genus *Pseudamnicola*. However, due to the characteristic shape of the penis and the tentacles it is transfered to *Sarkhia* gen. n.

##### Distribution.

Iran; Kermanshah and Markazi Provinces.

### Genus *Belgrandiella* Wagner, 1927

**Type species.** *Belgrandia kusceri*Wagner, 1914

#### Belgrandiella elburensis

(Starmühlner & Edlauer, 1957) comb. n.

http://species-id.net/wiki/Belgrandiella_elburensis

##### Records from Iran.

Tehran Province – “*Frauenfeldia elburensis*” Starmühlner and Edlauer (1957).

##### Remarks.

Starmühlner and Edlauer (1957) originally described this species as *Frauenfeldia elburensis*. However, the genus name *Frauenfeldia* is preoccupied, and thus, the species of this genus have been re-assigned to *Belgrandiella*, *Boleana*, *Graziana* and *Sarajana* (Radoman 1983). Due to the shape of the aperture in original description (see Starmühlner and Edlauer 1957) we affiliate this species to the genus *Belgrandiella*.

##### Distribution.

Iran, only known from the locus typicus (Gelandoah, 60 km NE of Tehran).

### Genus *Hauffenia* (Pollonera, 1898)

**Type species.** *Valvata erythropomatia* Hauffen, 1856

#### Hauffenia erythropomatia

(Hauffen, 1856)

http://species-id.net/wiki/Hauffenia_erythropomatia

##### Records from Iran.

Sistan and Baluchestan Province (Source lake Gomun) – “*Erythropomatiana erythropomatia*” Starmühlner and Edlauer (1957).

##### Remarks.

Most probably, Starmühlner and Edlauer (1957) misidentified this subterranean species, known only from its type locality in Slovenia, far away from Iran. The comparison with the description of *Hauffenia erythropomatia* by Radoman (1983) shows that these species are not conspecific as the umbilicus seems to be broader in later species compared with the species depicted by Starmühlner and Edlauer (1957). Unfortunately this species could not be found in Edlauer’s collection in NHMW (Anita Eschner, pers. comm.). The record for this species is kept until specimens from the original locality could be studied.

### Family Stenothyridae Tryon, 1866

Genus *Stenothyra* Benson, 1854

**Type species.** *Nematura deltae* Benson, 1836

#### Stenothyra arabica

Neubert, 1998

http://species-id.net/wiki/Stenothyra_arabica

##### Records from Iran.

Hormozgan Province (Ghasemi et al. 2011).

##### Distribution.

Saudi-Arabia, Iran.

### Genus *Gangetia* Ancey, 1890

**Type species.** *Hydrobia (Belgrandia) miliacea* Nevill, 1880

#### Gangetia (Iranothyra) uzielliana

(Issel, 1866)

http://species-id.net/wiki/Gangetia_uzielliana

##### Records from Iran.

Kerman province (as *Bythinia uzielliana*: Issel 1866,Martens 1874), as *Hydrobia uzielliana*:Biggs (1936, 1937), (as *Pseudamnicola uzelliana*:Starmühlner and Edlauer (1957), (as *Pseudamnicola uzelliana*: Starmühlner (1961, 1965); Fars province (as *Pseudamnicola uzelliana*: Starmühlner and Edlauer (1957), (as *Pseudamnicola uzelliana*: Starmühlner and Edlauer (1961, 1965).

##### Rejected records.

Yazd Province (as *Pseudamnicola uzelliana*: Starmühlner and Edlauer 1957).

##### Remarks.

Schütt (1973) classified this species in the genus *Gangetia* and introduced the new subgenus *Iranothyra* Schütt, 1973. Mansoorian (1994) reported *Gangetia uzielliana* with some doubts. However, his species clearly differs from the topotype of *Gangetia uzielliana* illustrated by Schütt (1973). Most probably, the species recorded by Mansoorian (1994) under this name represents an undescribed new species (Glöer and Pešić 2009).

##### Distribution.

Iran.

### Genus *Farsithyra* Glöer & Pešić, 2009

**Type species.** *Farsithyra farsensis* Glöer & Pešić, 2009

#### Farsithyra farsensis

Glöer & Pešić, 2009

http://species-id.net/wiki/Farsithyra_farsensis

Fig. 12f 13a–b

Bulimus badiella : Starmühlner and Edlauer 1957, non *badiella* Küster, 1852 (synonymy)

##### Records from Iran.

Fars Province (Glöer and Pešić 2009).

##### New records.

Hormozgan Province: IR17-11 [1 ex.].

##### Material examined.

NHMW“*Pseudamnicola uzelliana* Issel”, Persien, stark salziger Tümpel, südl.von Yest (=Yesd), leg.Starmühlner. NHMW 60.459 “*Bulimus badiella*“, Lake Taschk, 07.07.1956 leg. Löffler.

##### Associated species.

*Melanoides tuberculatus*, *Melanopsis* sp., *Melanopsis doriae*, *Thiara scabra*.

##### Remarks.

**Figure 13.**
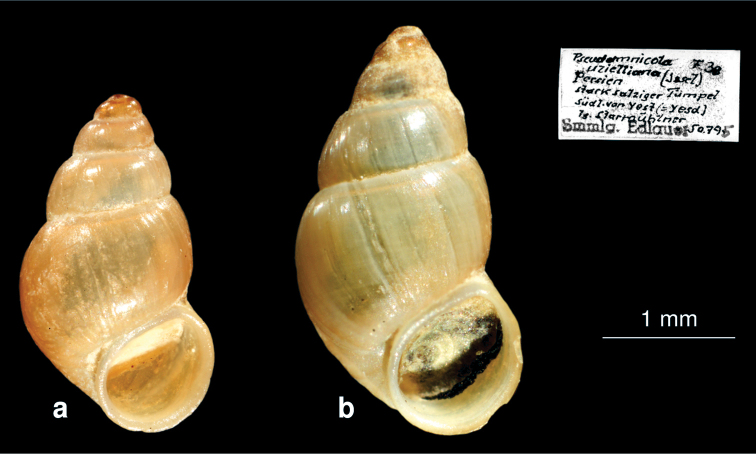
*Farsithyra farsensis* (from Edlauer’s collection, NHMW “*Pseudamnicola uzielliana*” 75000/E/50795): **a–b** shell.

Starmühlner and Edlauer (1957) mentioned *Gangetia uzielliana* from many sampling sites in Yazd Province. An analysis of one lot from the Edlauer collection (NHMW) with the specimens from Yazd Province shows that these specimens (Fig. 13a–b) belong to *Farsithyra farsensis*. Further, re-examination of the specimens from Lake Taschk in Fars Province identified by Starmühlner and Edlauer (1957) as *Bulimus badiella* (syn. to *Bithynia badiella*) shows that it is also conspecific with *Farsithyra farsensis*.

##### Distribution.

Iran: Fars, Yazd and Hormozgan Provinces.

### Family Valvatidae J.E. Gray, 1840

Genus *Valvata* O.F. Müller, 1773

**Type species.** *Valvata cristata* O.F. Müller, 1774

#### Valvata cristata

O.F. Müller, 1774

http://species-id.net/wiki/Valvata_cristata

Fig. 12m

##### New records.

Mazandaran Province: IR01-05 [6 ex.]. Tehran Province: IR48-05 [2 ex.].

##### Associated species.

*Bithynia mazandaranensis* sp. n.,*Planorbis carinatus*, *Anisus* sp.,* Valvata nowshahrensis* sp. n., *Hippeutis complanatus*.

##### Records from Iran.

Mansoorian (1994).

##### Remarks.

Considering the photo provided by Mansoorian (1994), he probably confused this species with *Valvata nowshahrensis* sp. n. (see below).

##### Distribution.

Palaearctic.

#### Valvata piscinalis

O.F. Müller, 1774

http://species-id.net/wiki/Valvata_piscinalis

##### Records from Iran.

Gilan, Mazandaran and Lorestan Province – Mansoorian (2000).

##### Distribution.

Palaearctic.

#### Valvata nowshahrensis sp. n.

urn:lsid:zoobank.org:act:944E6EE3-B23C-43FB-A305-882A4D4CF3D9

http://species-id.net/wiki/Valvata_nowshahrensis

Fig. 14a–c

##### Type locality.

Mazandaran Province, Nowshahr city, pond near the Caspian See, 51°31'E, 36°38'N, 18 June 2005.

##### Holotype

(ZMH 79376): Shell diameter 3.3 mm, height 2.3 mm.

##### Paratypes

(ZMH 79377): 2 specimens from type locality; [2 ex.], Kermanshah Province: IR105-05.

##### Etymology.

Named after the region, where the species was collected.

##### Description.

The yellowish shell is translucent with 3 circular whorls. The umbilicus is wide, and the first whorl is visible through the umbilicus. The surface is glossy with very fine ribs. Shell diameter 3.2–3.3 mm, height 2.3 mm.

##### Differentiating features.

The new species can be distinguished from *Valvata piscinalis* by its larger umbilicus and from *Valvata cristata* by its higher spire.

##### Remarks.

This species has possibly been depicted by Mansoorian (1994) and confused with *Valvata cristata*.

##### Associated species.

*Pseudobithynia mazandaranensis* sp. n.,*Planorbis carinatus*, *Anisus* sp., *Valvata cristata*, *Hippeutis complanatus*

##### Distribution.

Iran: Mazandaran and Kermanshah Provinces.

**Figure 14.**
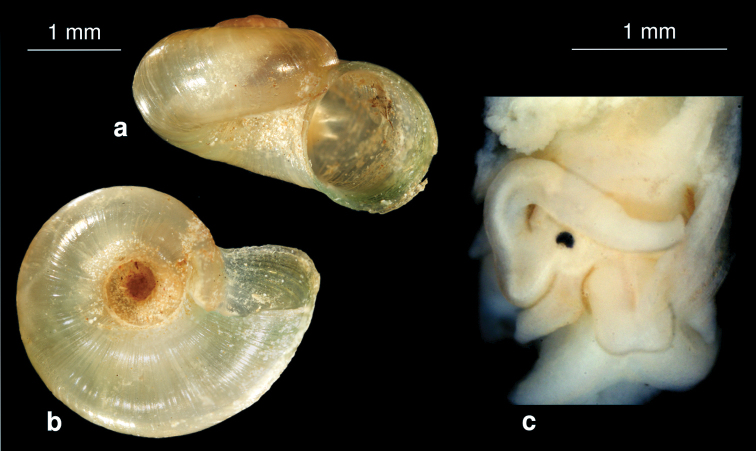
*Valvata nowshahrensis* sp. n. **a** shell **b** ventral view on the umbilicus **c** head with penis in situ.

### Pulmonata

Family Acroloxidae Thiele, 1931

Genus *Acroloxus* H. Beck, 1838

**Type species.** *Patella lacustris* Linnaeus, 1758

#### Acroloxus lacustris

(Linnaeus, 1758)

http://species-id.net/wiki/Acroloxus_lacustris

##### Rejected Records from Iran.

Mazandaran Province – Forcart (1935).

##### Remarks.

See remarks under *Acroloxus pseudolacustris* sp. n.

#### Acroloxus pseudolacustris sp. n.

urn:lsid:zoobank.org:act:83575F59-E417-44D3-8F6D-A5DB45EA2B21

http://species-id.net/wiki/Acroloxus_pseudolacustris

Fig. 15a–b

##### Type locality.

Gilan Province, IR82-05, Bandar Anzali Lagoon, 49°27'E, 37°26'N, 16 June 2008.

##### Holotype

(ZMH 79378): Shell length 4.0 mm, width 2.0 mm, height 0.9 mm.

##### Paratypes.

2 ex., NMB 11516a “*Acroloxus lacustris*” zwischen Nika und Aschref, 10 m ü. M., Drs. A. Erni & R. Buxtorf leg. 22.X.1931.

##### Etymology.

Named for its resemblance with *Acroloxus lacustris*.

##### Description.

**Figure 15.**
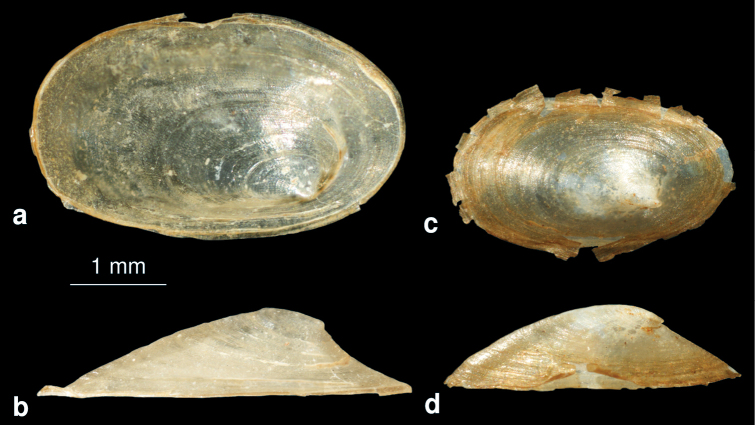
**a–b** *Acroloxus pseudolacustris* sp. n.: shell **c–d** *Acroloxus lacustris* (from Hamburg, Germany): shell.

The oval limpet shell is transparent. The apex is blunt and bent to the left side (Figs 15a–b).

##### Differentiating features.

The new species resembles *Acroloxus lacustris*, which can be easily distinguished by the shape of apex, which is always acute and not blunt (Figs 15c–d) like in the new species. From Russia, no *Acroloxus* sp. with a blunt apex is known (Vinarski, pers. comm.).

##### Remark.

An analysis of the two specimens from Forcart’s collection (NMB 11516a) identified as *Acroloxus lacustris* from Mazandaran Province shows that these specimens belong to *Acroloxus pseudolacustris* sp. n.

##### Associated species.

*Haitia acuta*.

##### Distribution.

Iran: Gilan and Mazandaran Provinces.

### Family Lymnaeidae Rafinesque, 1815

#### Genus Radix

Montfort, 1810

http://species-id.net/wiki/Radix

##### Type species.

*Helix auricularia* Linnaeus, 1758

##### Remarks.

Hubendick (1951) grouped most *Radix* spp. from the Near East (i.e. *Radix tenera*, *Radix euphratica* – «Mesopotamia», *Radix bactriana* - Afghanistan, *Radix gedrosiana* - Iran, *Radix rectilabrum* - Seistan and Baluchistan, *Radix persica* - Iran and *Radix acuminata* - (Bengal, India) under the palaearctic *Radix auricularia*, but from Europe he lumped all *Radix* spp. together in three species. Today, five *Radix* species are known from Europe, confirmed by molecular (Pfenninger et al. 2006, Schniebs et al. 2011) and anatomical studies (Glöer 2002). Only a few species can be distinguished by the shells alone (e.g. *Radix ampla*, *Radix auricularia*). Most species show a large morphological plasticity in the shape of the shell, so this character cannot be used for distinguishing species. Ananndale and Prashad (1919) and Annandale and Rao (1923) provided anatomical data of *Radix* spp., but these drawings are not suitable enough to identify the *Radix* spp. found by us in Iran. The diagnostic features and taxonomic relationship of the Iranian *Radix* species require further revision and particularly the application of molecular techniques with topotypes of the species. The following list of *Radix* species contains the hitherto recorded nominal species, their taxonomic status remains to be explored.

#### Radix persica

(Issel, 1865)

http://species-id.net/wiki/Radix_persica

Fig. 16a

##### Records from Iran.

Kerman Province – “*Limnaea auricularia* var. *persica”* Issel (1865), “*Limnaea auricularia* var. *persica”* Martens (1874); Seistan and Baluchestan Province (as *Limnaea auricularia* var. *persica*: Annandale and Prashad 1919); Isfahan Province (as *Lymnaea persica*:Biggs 1937).

##### New records.

Markazi Province:IR27-07 [7 ex.]

##### Distribution.

South Iran.

**Figure 16.**
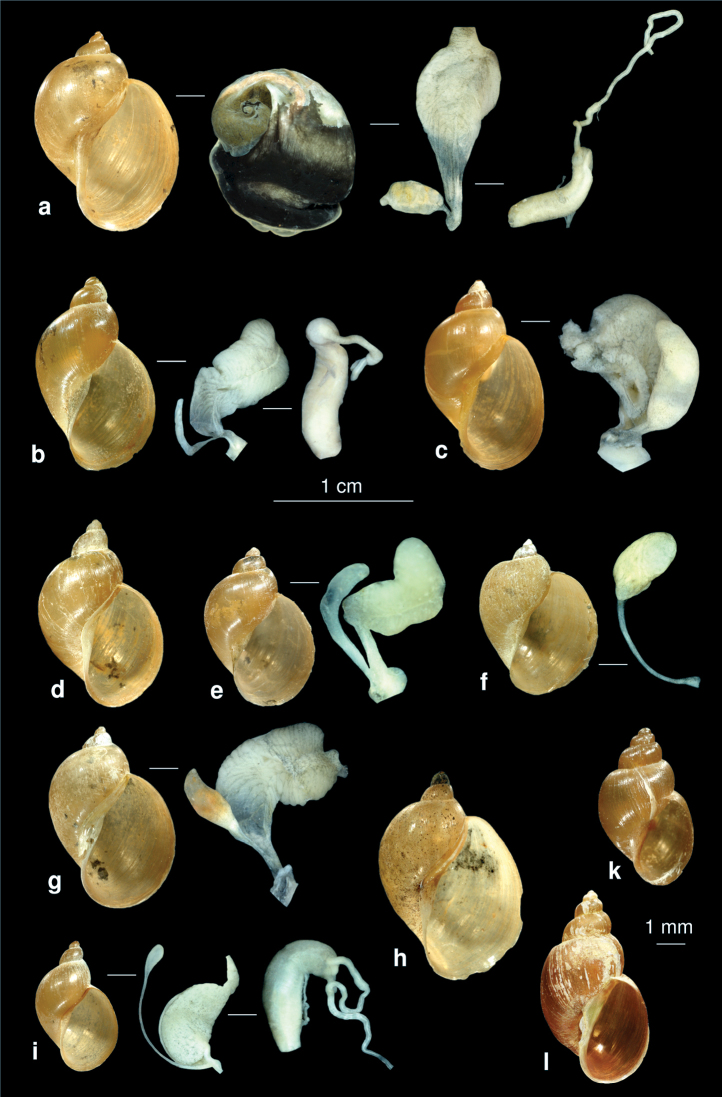
The Lymnaeidae of Iran. **a** *Radix persica* (IR27-07) **b–d** *Radix bactriana* (**b** IR03-05 **c** IR87-05 **d** IR88-05 **e** IR91-05) **f** *Radix persica* (IR107-05) **g** *Radix iranica* (IR89-05) **h** *Radix* sp. **i** *Radix* sp. **k** *Galba truncatula* (IR62-05) **l** *Galba schirazensis*.

#### Radix auricularia

(Linnaeus, 1758)

http://species-id.net/wiki/Radix_auricularia

##### Records from Iran.

Khuzestan Province (Mansoorian 2001); Mazandaran, Gilan and Lorestan Provinces (Starmühlner and Edlauer 1957), Isfahan Province (Starmühlner 1965).

##### Distribution.

Palaearctic.

#### Radix bactriana

(Annandale & Prashad, 1919)

http://species-id.net/wiki/Radix_bactriana

Figs 16b–d

##### Records from Iran.

Seistan and Baluchestan Province (Annandale and Prashad 1919); Kerman Province (Starmühlner and Edlauer 1957).

##### New records.

Markazi Province: IR03-05 [1 ex], IR87-05 [9 ex.], IR88-05 [3 ex.], IR89-05 [2 ex], IR91-05 [3 ex.]; Khorasan Province: IR67-05 [1 ex.], IR79-05 [1 ex.].

##### Distribution.

Iran:Seistan and Baluchestan and Kerman Provinces.

#### Radix iranica

(Annandale & Prashad, 1919)

http://species-id.net/wiki/Radix_iranica

Fig. 16g

##### Records from Iran.

Seistan and Baluchestan Province (Annandale and Prashad 1919).

##### New records.

Markazi Province:IR89-05 [5 ex].

##### Distribution.

Iran:Seistan and Baluchestan Province.

#### Radix gedrosiana gedrosiana

(Annandale & Prashad, 1919)

http://species-id.net/wiki/Radix_gedrosiana_gedrosiana

##### Records from Iran.

Seistan and Baluchistan Province (Annandale and Prashad 1919), Azarbayjan Province (Starmühlner and Edlauer 1957), Khuzestan Province (Chu et al. 1968, Massoud and Hedayeti-Far 1979, as *Lymnaea auricularia gedrosiana*: Mansoorian 2001), N Iran (Annandale 2000).

##### Distribution.

Iran, Pakistan.

#### Radix gedrosiana rectilabrum

http://species-id.net/wiki/Radix_gedrosiana_rectilabrum

##### Records from Iran.

Seistan and Baluchestan Province (Annandale and Prashad 1919); Isfahan Province (Starmühlner and Edlauer 1957).

##### Distribution.

Iran;endemic.

#### Radix hordeum

(Mousson, 1874)

http://species-id.net/wiki/Radix_hordeum

##### Records from Iran.

Seistan and Baluchestan Province (Annandale and Prashad 1919)

##### Distribution.

Iraq(Euphrates, as*Limnaea hordea*: Mousson 1874); Iran:Seistan and Baluchestan Province.

#### Radix lagotis

(Schrank, 1803)

http://species-id.net/wiki/Radix_lagotis

##### Records from Iran.

Qom, Tehran and Gilan Provinces (Martens 1874); Kerman Province (Biggs 1937).

##### Remarks.

This species has been described from the Danube (Germany) and most probably does not occur in Iran. According to Subba Rao (1989) *Radix lagotis* is a synonym of *Radix peregra* (syn. to *Radix labiata*). However, recently Schniebs et al. (2011) clearly showed that *Radix lagotis* and *Radix labiata* are distinct species.

##### Distribution.

Europe.

#### Radix labiata

(Rossmaessler, 1835)

http://species-id.net/wiki/Radix_labiata

##### Records from Iran.

(mentioned as *Radix peregra* f. *canalifera*): N Iran (Caspian Sea) (Eliazian et al. 1979); Kerman Province (Starmühlner and Edlauer (1957); Fars Province (Starmühlner and Edlauer 1957); Yazd Province (Starmühlner and Edlauer 1957); Kermanshah Province (Starmühlner and Edlauer 1957), Starmühlner (1965).

##### Remarks.

*Radix labiata* is a species which prefers springs and is distributed in M – and S Europe and the Balkans (Glöer 2002).

##### Distribution.

Europe.

### Genus *Galba* Schrank, 1803

**Type species.** *Galba truncatula*O.F. Müller, 1774

#### Galba truncatula

(O.F. Müller, 1774)

http://species-id.net/wiki/Galba_truncatula

Fig. 16k

##### Records from Iran.

Seistan and Baluchestan Province (as *Limnaea truncatula*: Annandale and Prashad 1919); North Iran (Caspian Sea) (as *Lymnaea truncatula*:Eliazian et al. 1979); Manzandaran Province (Forcart 1935); Gilan, Mazandaran and Lorestan Province (Mansoorian 2000); Kerman Province (Starmühlner and Edlauer 1957, Biggs 1937); Tehran Province (Starmühlner and Edlauer 1957); Khuzestan Province (Mansoorian 2001, Chu et al. 1968, Massoud and Hedayeti-Far 1979); Isfahan Province (Biggs 1937); Semnan Province (Starmühlner 1961); Hormozgan Province (Starmühlner 1965).

##### New records.

Khorasan Province: IR63-05 [22 ex.]; IR66a-05 [1 ex.]; IR77-05 [1 ex.].

##### Associated species.

*Radix sp.*, *Planorbis intermixtus*, *Physella acuta*.

##### Distribution.

Worldwide.

#### Galba schirazensis

Küster, 1862

http://species-id.net/wiki/Galba_schirazensis

Fig. 16l

##### Records from Iran.

Fars Province (Küster 1862); Gilan Province (Bargues et al. 2010).

##### Distribution.

Iran, Mediterranean, Central America (Bargues et al. 2011).

### Genus *Stagnicola* Jeffreys, 1830

**Type species.** *Buccinum palustre* O.F. Müller, 1774

#### Stagnicola palustris

(O.F. Müller, 1774)

http://species-id.net/wiki/Stagnicola_palustris

##### Records from Iran.

Kerman Province (Martens 1874); Isfahan Province (Martens 1874); Qazvin and E Azarbayjan Provinces (Starmühlner and Edlauer 1957); Gilan, Mazandaran and Lorestan Provinces (Eliazian et al. 1979); N Iran (Mansoorian 2000).

##### Rejected Records from Iran.

Mazandaran Province (Forcart 1935).

##### Remark.

The recent insights on the distribution of *Stagnicola palustris* show that it is a Northern European/Siberian species. Most probably, the species reported from Iran as *Stagnicola palustris* represents an undescribed species (see below).

#### Stagnicola sp.

Fig. 17

##### Records from Iran.

Mazandaran Province (Forcart 1935 ).

**Material examined:** 35 ex., NMB 11518b “*Stagnicola palustris*“Zw. Nika und Aschref, Dr. Erni & Buxtorf 1934; 3 ex., NMB 11518a “Iran, Prov. Mazandaran. Meschhediser, Geniste am rechten Ufer des Babul ca. 300 m S der Mündung, -26 m Meereshöhe. Leg. 23.8.1931 & don. 1935 Drs. A. Erni & R. Buxtorf”.

##### Remark.

**Figure 17.**
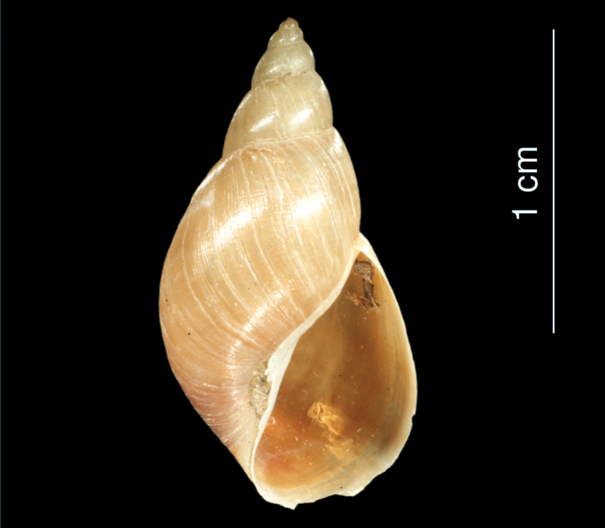
*Stagnicola* sp. (from Forcart’s collection, NMB 11518b “*Stagnicola palustris*”): shell.

An examination of the specimens from NMB identified by Forcart (1935) as *Stagnicola palustris* shows that these specimens are not conspecific with *Stagnicola palustris*. Namely, Forcart’s specimens clearly differ in the aperture, which is broader at the basis (Fig. 17) than in *Stagnicola palustris*. However, due to the fact that the shells of *Stagnicola* spp. are very variable, it is not possible to identify or eventually describe this specis as new to science without anatomical studies.

### Genus *Lymnaea* Lamarck, 1799

**Type species.** *Lymnaea stagnalis*(Linnaeus, 1758)

#### Lymnaea stagnalis

(Linnaeus, 1758)

http://species-id.net/wiki/Lymnaea_stagnalis

##### Records from Iran.

Khuzestan Province (Mansoorian 1998, 2001).

##### Distribution.

Palaearctic.

### Family Planorbidae Rafinesque, 1815

Genus *Bulinus* O.F. Müller, 1781

**Type species.** *Physa truncata* Audouin, 1827

#### Bulinus truncatus

(Audouin, 1827)

http://species-id.net/wiki/Bulinus_truncatus

##### Records from Iran.

Khuzestan Province (Chu et al. 1968, Massoud and Hedayeti-Far 1979, Mansoorian 1994, 2001); Gilan Province (Mansoorian 2000).

##### Distribution.

Tropical Africa, Arabian Peninsula, Iran.

### Genus *Planorbis* O.F. Müller, 1774

**Type species.** *Helix planorbis* Linnaeus, 1758

#### Planorbis intermixtus

Mousson, 1874

http://species-id.net/wiki/Planorbis_intermixtus

Fig. 18b

Planorbis subangulatus Philippi, 1844; *Planorbis persicus* Ancey, 1900 (synonymy)

##### Records from Iran.

Northern Iran (as *Planorbis planorbis*: Mansoorian 2000); Mazandaran Province (as *Planorbis planorbis*:Eliazian et al. 1979, Mansoorian 2000); Fars Province (as *Planorbis planorbis*: Forcart 1935, Starmühlner and Edlauer 1957); IsfahanProvince (Glöer and Pešić 2010); Yazd Province (as *Planorbis persicus*, *Planorbis subangulatus*: Biggs 1937, 1971, Starmühlner and Edlauer 1957); Gilan Province (as *Anisus (Gyraulus) intermixtus*:Starmühlner and Edlauer 1957); Khuzestan Province (as *Planorbis planorbis*, *Planorbis planorbis submarginatus*:Starmühlner and Edlauer 1957, as *Planorbis planorbis*:Biggs 1971); Markazi Province (Chu et al. 1968, Massoud and Hedayeti-Far 1979, Mansoorian 2001, Glöer and Pešić 2010).

##### New records.

Mazandaran Province: IR01-05 [11 ex.]; Markazi Province: IR51-05 [11 ex.]; IR87-05 [3 ex.]; IR88-05 [7 ex.]; IR91-05 [5 ex.]; IR93-05 [1 ex.]; Khorasan Province: IR66-05 [10 ex.]; IR67-05 [2 ex.]; IR68-05 [5 ex.]; IR78a-05 [2 ex.]; IR78b-05 [7 ex.]; Fars Province: IR02-07 [2 ex.]; IR07-07 [2 ex.]; IR26-07 [9 ex.]; IR27-07 [3 ex.].

##### Associated species.

*Physella acuta*, *Pseudobithynia zagrosia*, *Radix* sp.

##### Remarks.

The species *Planorbis planorbis* and *Planorbis intermixtus* can only be distinguished by the number of prostate diverticula (Glöer and Pešić 2010). All *Planorbis* spp. collected in Iran have been anatomically studied and no *Planorbis planorbis* could be found. Thus we list the old records from Iran under *Planorbis intermixtus*.

In addition, *Planorbis subangulatus* Philippi, 1844 and *Planorbis persicus* Ancey, 1900 have been mentioned from Iran (Ancey 1900, Biggs 1937). Both species have been described on the basis of the shells, the morphology of which falls within variability of *Planorbis intermixtus*. Thus we list these species under *Planorbis intermixtus*.

##### Distribution.

Turkey, Iran, N India.

**Figure 18.**
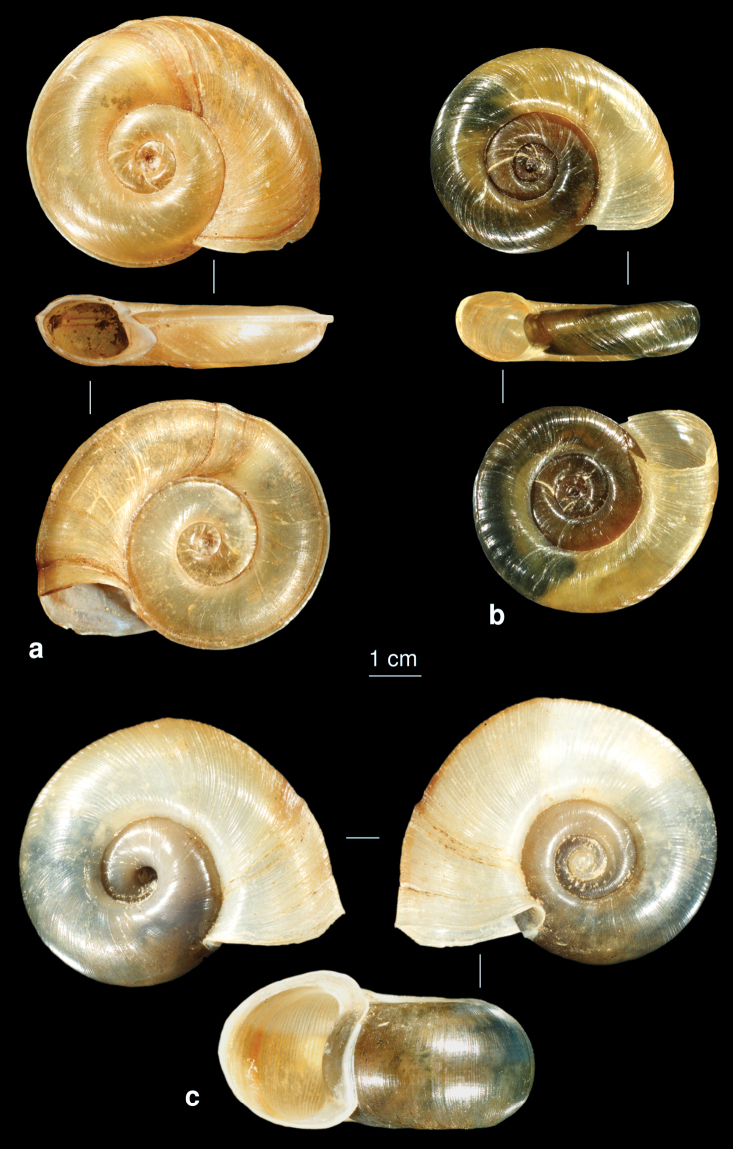
The *Planorbis* spp. of Iran. **a** *Planorbis carinatus* **b** *Planorbis intermixtus* **c** *Indoplanorbis exustus*.

#### Planorbis carinatus

O.F. Müller, 1774

http://species-id.net/wiki/Planorbis_carinatus

Fig. 18a

##### Records from Iran.

Northern Iran (Mansoorian 1994).

##### New records.

Mazandaran Province:IR01-05 [5 ex., anat. det. ].

##### Associated species.

*Valvata cristata*, *Anisus* sp., *Valvata nowshahrensis* sp. n., *Pseudobithynia mazandaranensis* sp. n., *Hippeutis complanatus*.

##### Distribution.

Palaearctic.

#### Genus Anisus

S. Studer, 1820

http://species-id.net/wiki/Anisus

##### Type species.

*Helix spirorbis* Linnaeus, 1758

##### Remarks.

The identification of the species of this genus is based on the anatomical features (Glöer and Meier-Brook 2008), so all former records of this genus are questionable and need new confirmation.

#### Anisus leucostoma

(Millet, 1813)

http://species-id.net/wiki/Anisus_leucostoma

##### Records from Iran.

Gilan Province – Mansoorian (1994, 2000).

##### Distribution.

Palaearctic.

#### Anisus spirorbis

(Linnaeus, 1758)

http://species-id.net/wiki/Anisus_spirorbis

##### Records from Iran.

Azarbayjan Province (Starmühlner and Edlauer 1957)

##### Distribution.

Palaearctic.

#### Anisus sp.

Fig. 19

##### New records.

Mazandaran Province: IR01-05 [1 empty shell].

##### Associated species.

*Planorbis carinatus*, *Anisus* sp., *Valvata nowshahrensis* sp. n.,* Pseudobithynia mazandaranensis* sp. n., *Hippeutis complanatus*.

##### Remarks.

**Figure 19.**
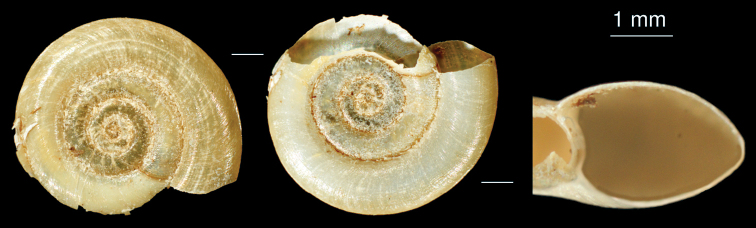
*Anisus* sp.: shell.

The shells (Fig. 19) of this species are similar to the rare species *Anisus vorticulus*, which is distributed in Central and E Europe. Additional material is necessary to resolve the taxonomy of this taxon.

#### Anisus vortex

(Linnaeus, 1758)

http://species-id.net/wiki/Anisus_vortex

##### Records from Iran.

Fars Province – Mansoorian (1994).

##### Distribution.

Euro-Siberian.

### Genus *Gyraulus* Charpentier, 1837

**Type species.** *Planorbis albus* O.F. Müller 1774

#### Gyraulus piscinarum

(Bourguignat, 1852)

http://species-id.net/wiki/Gyraulus_piscinarum

Fig. 20b

##### Records from Iran.

Tehran Province (as *Anisus (Gyraulus) piscinarum*: Starmühlner and Edlauer 1957).

##### New records.

Mazandaran Province: IR02-05 [6 ex.]; Fars Province: IR07-07 [13 ex.]; Seistan and Baluchestan Province: IR08-11 [10 ex.], IR09-11 [14 ex.].

##### Distribution.

Lebanon, Syria, Turkey (Black Sea coast), Iran.

##### Remark.

The examined specimens have been identified by its anatomy and are in a good agreement with Glöer and Bößneck (2007) as well as the anatomical studies carried out by Meier-Brook (1983).

**Figure 20.**
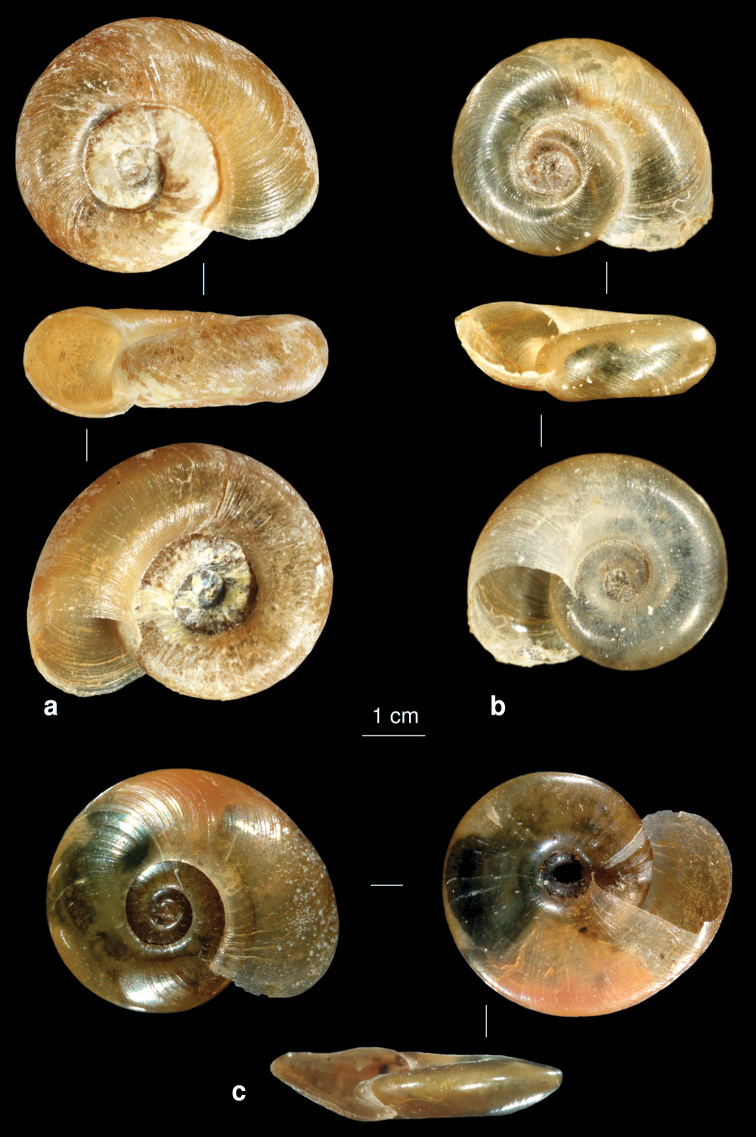
**a** *Gyraulus convexiusculus* **b** *Gyraulus piscinarum* **c** *Hippeutis complanatus*.

#### Gyraulus euphraticus

(Mousson, 1874)

http://species-id.net/wiki/Gyraulus_euphraticus

##### Records from Iran.

Seistan and Baluchestan Province (Annandale and Prashad 1919); Fars Province (Starmühlner and Edlauer 1957); Khuzestan Province (Massoud and Hedayeti-Far 1979, Mansoorian 2001).

##### Remarks.

*Gyraulus euphraticus* can be confused with *Anisus* spp. (Glöer and Bössneck 2007).

##### Distribution.

Irak, Iran.

#### Gyraulus convexiusculus

(Hutton, 1849)

http://species-id.net/wiki/Gyraulus_convexiusculus

Fig. 20a

##### New records.

Seistan and Baluchestan Province: IR08-05 [2 ex.]; IR09-11 [4 ex.].

##### Records from Iran.

Seistan and Baluchestan Province (Annandale and Prashad 1919); Yazd Province (Starmühlner and Edlauer 1957).

##### Distribution.

Afghanistan to Thailand, Iran.

#### Gyraulus laevis

(Alder, 1838)

http://species-id.net/wiki/Gyraulus_laevis

##### Records from Iran.

Mazandaran Province (Forcart 1935, Starmühlner and Edlauer 1957).

##### Distribution.

Central Europe.

### Genus *Indoplanorbis* Annandale and Prashad, 1920

**Type species.** *Planorbis exustus*Deshayes, 1834

#### Indoplanorbis exustus

(Deshayes, 1834)

http://species-id.net/wiki/Indoplanorbis_exustus

Fig. 18c

##### Records from Iran.

Seistan and Baluchestan Province (Mansoorian 1994).

##### New records.

Hormozgan Province: IR15-11 [5 ex.].

##### Distribution.

Iran, Oman, Yemen, India, Nepal, SE Asia.

### Genus *Hippeutis* Charpentier, 1837

**Type species.** *Helix complanata* Linnaeus, 1758

#### Hippeutis complanatus

(Linnaeus, 1758)

http://species-id.net/wiki/Hippeutis_complanatus

Fig. 20c

##### New records.

Mazandaran Province: IR01-05 [3 ex., anat. det.].

##### Remarks.

New for Iran.

##### Distribution.

Europe to W Asia.

### Genus *Segmentina* Fleming, 1818

**Type species.** *Planorbis nitidus* O.F. Müller, 1774

#### Segmentina calatha

(Benson, 1850)

http://species-id.net/wiki/Segmentina_calatha

##### Records from Iran.

Seistan and Baluchestan Province (Annandale and Prashad 1919).

##### Distribution.

India, Iran.

### Genus *Ferrissia* Walker, 1903

**Type species.** *Ferrissia rivularis* (Say, 1817)

#### Ferrissia isseli

(Bourguignat, 1866)

http://species-id.net/wiki/Ferrissia_isseli

##### Records from Iran.

Gilan Province (as *Protancylus (Ferrissia) isseli*: Starmühlner and Edlauer 1957).

##### Distribution.

Africa, Iran.

### Family Physidae Fitzinger, 1833

Genus *Haitia* Clench & Aguayo, 1932

**Type species.** *Physa globosa* Haldeman, 1841

#### Haitia acuta

(Draparnaud, 1805)

http://species-id.net/wiki/Haitia_acuta

##### Records from Iran.

all mentioned as *Physa acuta*: Gilan, Mazandaran and Lorestan Provinces (Mansoorian 2000); Khuzestan Province (Mansoorian 2001, Massoud and Hedayeti-Far 1979, Elazian et al. 1979).

##### New records.

Mazandaran Province: IR02-05 [2 ex.]; IR03-05 [3 ex.]; IR04-05 [3 ex.]; IR05-05 [3 ex.]; Markazi Province: IR51-05 [2 ex.], IR91-05 [6 ex.]; IR93-05 [1 ex.]; Khorasan Province: IR70-05 [1 ex.]; IR77-05 [1 ex.]; Gilan Province: IR82-05 [1 ex.]; Lorestan Province; IR95-05 [39 ex.]; Fars Province: IR07-07 [22 ex.]; IR14-07[13 ex.]; IR26-07 [3 ex]; Seistan and Baluchestan Province: IR08-11 [12 ex.]; Hormozgan Province. IR17-11 [1 ex.].

##### Associated species.

*Melanoides tuberculatus*, *Thiara scabra*, *Grossuana* sp., *Galba truncatula*, *Acroloxus pseudolacustris*, *Planorbis intermixtus*, *Pseudobithynia zagrosia*.

##### Distribution.

Europe,Mediterranean, Iraq, Iran.

### Discussion

The checklist of Iranian freshwater snails presented here shows a total of 73 species in 34 genera and 14 families. The records and taxonomic status of six species i.e. *Neritina mesopotamica* Martens, 1874, *Bithynia* cf. *ejecta* Mousson, 1874, *Bithynia rubens* (Menke, 1830), *Hydrobia acuta* (Draparnaud, 1805), *Hauffenia erythropomatia* (Hauffen, 1856) and *Stagnicola palustris* (O.F. Müller, 1774) are questionable and needs new confirmation. Further, the genus *Melanopsis* needs revision as several species have been reported from Iran (i.e., *Melanopsis variabilis*, *Melanopsis deserticola*, *Melanopsis buccinoidea* and *Melanopsis praemorsa*), but without further study and additional materials it is not possible to establish under which name or names the Iranian populations should be placed. The genus *Radix* is richest in the number of the species. However, our list of *Radix* species from Iran contains the hitherto recorded nominal species, their taxonomic status remains to be explored. For the two species i.e. *Stagnicola* sp. and *Anisus* sp. further study and additional specimens are necessary to resolve the taxonomy of these taxa. The identification of the species of the genus *Anisus* is based on the anatomical features (Glöer and Meier-Brook 2008), so all former records of this genus are questionable and need new confirmation. Three species, *Bithynia badiella* (Küster, 1852), *Bithynia troschelii* (Pasch, 1842) and *Acroloxus lacustris*(Linnaeus, 1758), are excluded from the list of Iranian freshwater snails, while *Bithynia tentaculata* most probably does not occur in Iran.

Of the 73 species reported in this paper, 12 species have a wide distribution (known from two or more bieogeographical regions), 9 species are Palaearctic, 4 species are W-Palaearctic and 8 species are “Middle East” (Iran, Iraq, Tadjikistan, Uzbekistan, Turkey, Syria, Israel) in their distribution. Insufficient knowledge hampers the determination of the biogeographic status of the rest of the species. Moreover, another 27 (37%) of these species have been indicated as being endemic to Iran.

If we take generic diversity into consideration, we can see that only three genera i.e. *Farsithyra* Glöer & Pešić 2009, *Kaskakia* gen. n.and *Sarkhia* gen. n. are endemic to Iran.

The species-richness of freshwater gastropods in our study was rather low one with an average of 2.12 species and a maximum of 6 spp. per sampling site. Only some common species occur in high abundances [> 20 ind./sampling site], abundances of most species being < 10 ind./sampling site. Most sampling sites in our study were intermittent streams, with perennial surface water only present in the head water section near their source in the mountains. Further downstream, riverbeds are usually seasonally dry with occasionally some standing pools in their middle course (Pešić et al. 2012).

As expected, our current knowledge of the diversity of the freshwater snail fauna is far from being complete. For most Iranian provinces, all available data come from a few surveys with as objective the study of snails as vectors of digenetic trematodes of medical or veterinary importance (e.g., Mansoorian 1994, 1998, 2001). However, large portions of Iran remain unexplored and many important hydrological basins have never been sampled. The number of known species may hence only represent but a part of the total freshwater snail species number in Iran. For example, for Central Europe, an estimated total species number of about 150 appears appropriate (Glöer 2002).

However, the present study is exhaustive and constitutes the most complete list of freshwater snails in Iran, including a complete bibliography of research on the subject. Further studies should focus at a serious improvement of our knowledge on Iranian freshwater snails by intensive collecting activities in little known areas in order to close the large gaps in our knowledge on their diversity. Particularly some specific habitats such as springs and underground habitats are more or less unexplored but may prove to be a major source for freshwater biodiversity.

## Supplementary Material

XML Treatment for Neritidae

XML Treatment for Neritina mesopotamica

XML Treatment for Neritina cinctellus

XML Treatment for Neritina euphratica

XML Treatment for Theodoxus fluviatilis

XML Treatment for Theodoxus lituratus

XML Treatment for Theodoxus pallida

XML Treatment for Bellamya bengalensis

XML Treatment for Bellamya hilmandensis

XML Treatment for Melanopsis

XML Treatment for Melanopsis costata

XML Treatment for Melanopsis doriae

XML Treatment for Melanopsis kotschyi

XML Treatment for Melanopsis

XML Treatment for Cerithidea cingulata

XML Treatment for Thiara scabra

XML Treatment for Melanoides tuberculatus

XML Treatment for Bithynia (Bithynia) tentaculata

XML Treatment for Bithynia (Bithynia) forcarti

XML Treatment for Bithynia (Bithynia) starmuehlneri

XML Treatment for Bithynia (Bithynia) mazandaranensis

XML Treatment for Bithynia (Bithynia) ejecta

XML Treatment for Bithynia (Bithynia) rubens

XML Treatment for Gabbia

XML Treatment for Bithynia (Gabbia) sistanica

XML Treatment for Pseudobithynia irana

XML Treatment for Pseudobithynia zagrosia

XML Treatment for Heleobia dalmatica

XML Treatment for Hydrobia acuta

XML Treatment for Ecrobia grimmi

XML Treatment for Pseudamnicola kotschyi

XML Treatment for Pseudamnicola saboori

XML Treatment for Pseudamnicola zagrosensis

XML Treatment for Pseudamnicola raddei

XML Treatment for Pseudamnicola georgiev

XML Treatment for Kaskakia

XML Treatment for Kaskakia khorrasanensis

XML Treatment for Sarkhia

XML Treatment for Sarkhia sarabensis

XML Treatment for Sarkhia kermanshahensis

XML Treatment for Belgrandiella elburensis

XML Treatment for Hauffenia erythropomatia

XML Treatment for Stenothyra arabica

XML Treatment for Gangetia (Iranothyra) uzielliana

XML Treatment for Farsithyra farsensis

XML Treatment for Valvata cristata

XML Treatment for Valvata piscinalis

XML Treatment for Valvata nowshahrensis

XML Treatment for Acroloxus lacustris

XML Treatment for Acroloxus pseudolacustris

XML Treatment for Radix

XML Treatment for Radix persica

XML Treatment for Radix auricularia

XML Treatment for Radix bactriana

XML Treatment for Radix iranica

XML Treatment for Radix gedrosiana gedrosiana

XML Treatment for Radix gedrosiana rectilabrum

XML Treatment for Radix hordeum

XML Treatment for Radix lagotis

XML Treatment for Radix labiata

XML Treatment for Galba truncatula

XML Treatment for Galba schirazensis

XML Treatment for Stagnicola palustris

XML Treatment for Stagnicola

XML Treatment for Lymnaea stagnalis

XML Treatment for Bulinus truncatus

XML Treatment for Planorbis intermixtus

XML Treatment for Planorbis carinatus

XML Treatment for Anisus

XML Treatment for Anisus leucostoma

XML Treatment for Anisus spirorbis

XML Treatment for Anisus

XML Treatment for Anisus vortex

XML Treatment for Gyraulus piscinarum

XML Treatment for Gyraulus euphraticus

XML Treatment for Gyraulus convexiusculus

XML Treatment for Gyraulus laevis

XML Treatment for Indoplanorbis exustus

XML Treatment for Hippeutis complanatus

XML Treatment for Segmentina calatha

XML Treatment for Ferrissia isseli

XML Treatment for Haitia acuta

## Appendix

List ofsampling sites.

**Table:**

	**code**	**sampling site**	**GPS-coordinates [m asl.]**	**habitat**	**date**
1	IR01-05	Mazandaran province, Nowshahr city (near Caspian Sea)	51°31'E, 36°38'N [- 28]	pond	18.06.2005
2	IR02-05	Mazandaran province, road to Kandelous	no coordinates	spring	18.06.2005
3	IR03-05	Markazi province, road to Khomeyn	no coordinates	stream	27.06.2005
4	IR05-05	Lorestan province, Khorramabad area.	no coordinates	stream	24.06.2005
5	IR04-05	Khorasan province, 25 km to Bojnurd River	no coordinates	stream	07.07.2005
6	IR51-05	Markazi Province, Bolagh stream 10 km after Shahzand city (in Shahzand to Pole Doab Road),	no coordinates	stream	04.06.2005
7	IR61-05	Khorrasan Province, Akhlamad	58°57'E, 36°40'N [ca. 2000]	waterfall	04.06.2005
8	IR62-05	Khorrasan Province, Golmakan, Cheshmeh Sebz	59°15'E, 36°15'N [ca. 2000]	spring	04.06.2005
9	IR63-05	Khorrasan Province, Gojki road to Kalat (ca. 94 km to Kalat)	59°45'E, 36°35'N [ca. 1400 ]	rheohelocrenic spring	05.06.2005
10	IR64-05	Khorrasan Province, Gojki road to Kalat (ca. 94 km to Kalat)	59°45'E, 36°35'N [ca. 1400]	stream	05.06.2005
11	IR66a-05	Khorrasan Province, spring at Masshad-Kalat road (35 km to Kalat)	no coordinates	spring	05.06.2005
12	IR67-05	Khorrasan Province, river near Kalat city	59°45'E, 36°58'N [ca. 1900]	river	05.06.2005
13	IR68-05	Khorrasan Province, Mach stream (in Moghan road), 16 km to Moghan	59°31'E, 35°10'N [ca. 2000]	stream	06.06.2005
14	IR70-05	Khorrasan Province, Kaskak stream in Kaskak village	59°10'E, 35°25'N [ca. 1800]	stream	07.06.2005
15	IR76-05	Khorrasan Province, Kharv stream in Kharv city (25 km to Neishabour)	59°5'E, 36°12'N [ca. 2200]	stream	10.06.2005
16	IR77-05	Khorrasan Province, Koh Sorkh stream (in Koh Sorkh city, ca. 35 km to Kashmar city)	59°25'E, 36°25'N [ca. 2200]	stream	10.06.2005
17	IR78-05	Khorrasan Province, Zou Eram spring in Zou Eram village (near Shirvan city)	57°40'E, 37°20'N [ca. 1600]	spring	11.06.2005
18	IR78a-05	Khorrasan Province, spring 1 near Zou Eram spring in Zou Eram village (near Shirvan city)	no coordinates [ca. 1600]	spring	11.06.2005
19	IR78b-05	Khorrasan Province, spring 2 near to Zou Eram spring in Zou Eram village (near Shirvan city)	no ccordiantes [ca. 1600]	spring	11.06.2005
20	IR79-05	Khorrasan Province, Baba Aman Park spring (ca. 5 km to Bojnurd city)	57°24'E, 37°25'N [ca. 1300]	spring	11.06.2005
21	IR82-05	Gilan Province, Bandar Anzali Lagoon	49°27'E, 37°26'N	wetland	16.06.2005
22	IR87-05	Markazi Province, Ashtian to Arak road (ca. 5 km after Ashtian)	50°01'E, 34°34'N [ca. 1800]	pool	21.06.2005
23	IR88-05	Markazi Province Aman Abad spring in Anjedan road before Aman Abad village (ca. 5 km to Aman Abad village)	49°48'E, 33°55'N [ca. 1700]	spring	22.06.2005
24	IR89-05	Markazi Province, Cheshmeh Shater in Arak to Khomein road (8 km after Arak)	49°45'E, 34°08'N [ca. 1700]	pool	22.06.2005
25	IR90-05	Markazi Province, stream 2 km after Hassan Abad (in Arak to Khomein road)	49°52'E, 33°50'N [ca. 1700]	stream	22.06.2005
26	IR91-05	Markazi Province, Varcheh spring in Emamzadeh Varcheh village (in Arak to Khomein road, ca. 20 km to Khomein)	49°55'E, 33°49'N [ca. 1700]	spring	22.06.2005
27	IR93-05	Markazi Province, stream near Astaneh city (Azna Aligudarz cross way)	49°24'E, 33°55'N [ca. 2400]	stream	23.06.2005
28	IR94-05	Lorestan Province, Darband stream in Darband village (Azna to Dorood road, ca. 16 km to Azna)	49°17'E, 33°25'N [ca. 1800]	stream	23.06.2005
29	IR95-05	Lorestan Province, Dareh Takht stream in Dareh Takht village (13 km to Azna city)	33°22'N, 49°22'E [ca. 2800]	stream	23.06.2005
30	IR105-05	Kermanshah Province, Firoozan stream in Firoozan village	34°25'N, 48°11'E	stream	27.06.2005
31	IR106-05	Kermanshah Province, Sar Pol Kangarar stream in Sar Pol Kangarar village	34°30'N, 47°55'E	stream	27.06.2005
32	IR107-05	Kermanshah Province, spring near Sarabe – Sarkh city	no coordinates	spring	27.06.2005
33	IR108-05	Kermanshah Province, stream Gamasiab in village Gamasiab	34°27'N, 47°45'E	stream	27.06.2005
34	IR07-07	Fars Province, Dasht Arzhan village (in Shiraz to Kazerum road)	29°39'N, 51°59'E [ca. 2300]	stream	04.08.2007
35	IR13-07	Fars Province, AtashKadeh spring, Ardeshir palace in Firooz Abad	28°54'N, 52°32'E [1683]	limnocrenic spring	05.08.2007
36	IR14-07	Fars Province, Firooz Abad city, Kay Zarrin village	28°53'N, 52°32'E [1711]	stream	05.08.2007
37	IR17-07	Fars Province, Shiraz to Firooz Abad road, Ebrahim Abad village, Ebrahim Abad stream	29°00'N, 52°34'E	stream	06.08.2007
38	IR21-07	Lorestan Province, Mode Abad village	33°35''N, 48°37'E [1723]	stream	10.08.2007
39	IR26-07	Lorestan Province, road from Boroujerd to Khorram Abad city	33°30'N, 48°44'E [1660]	limnocrenic spring	10.08.2007
40	IR27-07	Markazi Province, Aman Abad (near Arak city)	33°59'N, 49°52'E [1775]	pool	11.08.2007
41	IR08-11	Seistan Province, Chabahar, Sharak village, Qanat 1	26°02'N, 61°04'E [264]	qanat (spring)	13.07.2011
42	IR08a-11	Seistan va Baluchestan Province, Chabahar, Sharak village, Qanat 2 (ca. 100 m from Qanat 1)	no coordinates	qanat (spring)	13.07.2011
43	IR09-11	Seistan va Baluchestan Province, Chabahar, Shirgovaz – Machkor stream, 45 m asl.	25°47'N, 61°28'E	stream	14.07.2011
44	IR10-11	Seistan va Baluchestan Province, Chabahar, Hootgat Bala river	25°48'N, 61°31'E [57]	river	14.07.2011
45	IR14-11	Hormozgan Province, Bandar Abass, Khorgoo village before hot water spring	27°29'N, 56°28'E [125]	saline stream	14.07.2011
46	IR15-11	Hormozgan Province, Bandar Abass, Khorgoo village before hot water spring, small pool near 14-11	27°29'N, 56°28'E [113]	pool (saline water)	16.07.2011
47	IR17-11	Hormozgan Province, Bandar Abass, Siahoo Qanat in Siahoo village	27°46'N, 56°20'E [630]	qanat (spring)	18.07.2011
48	IR18-11	Hormozgan Province, Bandar Abass, Taleguerdoo village, Poshtekeno spring, upper part of stream	27°49'N, 56°24'E [836]	stream	18.07.2011
49	IR19-11	Hormozgan Province, Bandar Abass, Banglayan village	27°46'N, 56°32'E [577]	stream	18.07.2011
50	IR20-11	Hormozgan Province, Bandar Abass, Bandar Kamir to Bandar Lenhueh road, ca 80 km to Bandar Abass, saline stream near Dezhgan	26°53'N, 55°16'E [20]	saline stream	20.07.2011
51	IR48-05	Tehran Province, Elbrus Mt., Shahrestanak River		river	18.08.2003
